# Altered metabolomics and inflammatory transcriptomics in human bone marrow adipocytes after acute high calorie diet and acute fasting

**DOI:** 10.3389/fendo.2025.1591280

**Published:** 2025-06-16

**Authors:** Samantha N. Costa, Gisela Pachón-Peña, Julie Dragon, Scott Tighe, Calvin Vary, Pouneh K. Fazeli, Miriam A. Bredella, Clifford J. Rosen

**Affiliations:** ^1^ Center of Molecular Medicine, MaineHealth Institute for Research, Scarborough, ME, United States; ^2^ Graduate School of Biomedical Science and Engineering, University of Maine, Orono, ME, United States; ^3^ Vermont Integrative Genomics Resources, University of Vermont, Burlington, VT, United States; ^4^ Division of Endocrinology, University of Pittsburgh School of Medicine, Pittsburgh, PA, United States; ^5^ Department of Radiology, Massachusetts General Hospital and Harvard Medical School, Boston, MA, United States; ^6^ Department of Radiology, New York University (NYU) Langone Health and Grossman School of Medicine, New York, NY, United States; ^7^ Center for Clinical and Translational Research, MaineHealth Institute for Research, Scarborough, ME, United States

**Keywords:** acute high calorie diet, acute fasting, RNA-sequencing, proteomics and lipidomics, immunosuppressive BM adipocytes, inflammatory response, lipid metabolism

## Abstract

Expansion of bone marrow (BM) adipocytes has been linked to nutritional pressures, suggesting that BM is a dynamic compartment that responds to fluctuations in systemic nutritional availability to regulate osteogenesis and hematopoiesis. Here, we investigated BM metabolism in response to acute overnutrition (high calorie diet; HCD) and calorie deprivation (fasting). Participants underwent a 10-day HCD followed by a two-week interval of an ad libitum diet and then underwent 10 days of fasting. BM adipocytes and sera were collected before and after each dietary intervention for each participant. Using comprehensive and integrated analyses, we characterized nutritional influences on BM adiposity. BM adipocytes after HCD showed an upregulation of *FOXP3*, the transcription factor that controls the development of Tregs, which are critical in reducing inflammatory immune responses. After fasting, BM adipocytes had an upregulation of inflammatory genes (*CP*, *CFH, VCAN*, and *IGFBP3)*. Proteomic analysis after HCD showed that BM serum had an upregulation of proteins related to an inflammatory/complement pathway. After fasting, in the BM serum, there was a significant downregulation of inflammatory/complement pathway proteins. Despite both interventions causing BM adipose tissue expansion, the mechanism for adipogenesis appears to be dependent on nutrient availability. After HCD, lipid-mediated signaling and lipid storage, and lipid droplet biogenesis were significantly downregulated. In contrast, after fasting, lipid-mediated signaling and lipid storage, and lipid droplet biogenesis were significantly upregulated. Overall, our results demonstrate key differences in inflammatory response and lipid metabolism between HCD and fasting, despite a nearly identical BM adipose phenotype. Further analyses are needed to understand the effects nutritional pressures have on BM adipogenesis and immune responses.

## 1 Introduction

Bone marrow adipose tissue (BMAT) is mostly found in the medullary canal of the long bones (tibia, femur, and humerus), in the vertebrae, and iliac crest (1–3). Bone marrow (BM) adipocytes, like osteoblasts, differentiate from skeletal stem cells (SSCs). BM is a heterogeneous population that includes skeletal and hematopoietic stem cells, mature immune cells, and non-lipid-laden preadipocytes. There is evidence that BMAT maintains skeletal homeostasis and influences whole-body energy metabolism (4, 5). Histologically, BM adipocytes resemble white adipocytes with a unilocular cell that contains a single lipid droplet. Unlike other fat depots, BMAT has a distinct metabolic and lipid composition that has local and systemic effects (6). The lipid content of BMAT, which is composed of saturated, monounsaturated, and polyunsaturated fats, can be used as an energy source for populations of osteoblasts, osteoclasts, and hematopoietic cells (7).

High calorie diet (HCD), calorie restriction, and calorie deprivation, such as fasting and models of anorexia nervosa, cause dramatic increases in BMAT in rodents and humans (8–11). Human studies of calorie deprivation have focused on patients with anorexia nervosa since peripheral fat depots are significantly reduced, but BMAT is increased to such an extent that it comprises up to 31% of total body adipose tissue (12). Research suggests that increased BMAT during calorie restriction or calorie deprivation is the result of adipogenesis with an increase in adipocyte number (i.e., hyperplasia) rather than an increase in adipocyte size (i.e., hypertrophy), whereas a HCD results in an overall increase in BM adiposity (number and size) (13). These differences may cause distinct alterations in BMAT function and energy expenditure in HCD compared to calorie restriction models (2). Several studies have identified BMAT as an endocrine organ with endocrine, paracrine, and autocrine signaling through the secretion of adipokines (Lep, IL6, TNFα, Mcp-1, Resistin, RANKL, Adipsin, Adipoq, RBP4) (1, 3, 14, 15). The adipokine secretion with BMAT expansion has also been shown to have immunomodulatory properties that affect BM myelopoiesis. People living with obesity have increased circulating monocyte populations, while fasting reduces the circulating pool of monocytes (16–18). This further demonstrates that changes in the BM (which is considered an immune regulatory organ), specifically BMAT expansion and adipokine secretion, can affect peripheral tissues in a manner that is dependent on the dietary intervention. Adipokine dysregulation with HCD/obesity and fasting/anorexia nervosa can contribute to associated co-morbidities, but the mechanistic aspects of BMAT development and maturation with dietary interventions are unknown.

Human BM adipocytes are also thought to respond to environmental cues, such as nutritional challenges (i.e., HCD or fasting) (19, 20). In our previous study, we analyzed BMAT changes in humans in response to weight gain with an acute HCD followed by weight loss with acute fasting (21). Our findings suggested that BMAT has a distinct behavior after HCD and fasting; however, in both dietary interventions, BMAT was increased (21). Using targeted RT-qPCR, we found that BMAT isolated after HCD had a proinflammatory response with increased TNF-α, Plexin D1, and SEMA3E gene expression. Similarly, there was an increase in BM serum resistin, a putative measure of macrophage activation. Previously, we postulated that BM adipocytes, much like peripheral adipose depots after HCD, are accompanied by an inflammatory response, including a rise in BM serum resistin, that could contribute to the long-term adverse sequelae of HCD, such as metabolic syndrome (10). In contrast, after fasting, our targeted analysis found no markers of inflammation in BMAT. However, our targeted approach limited our understanding of the local and systemic effects of BMAT accrual after dietary interventions.

Advancements in multi-omics studies (RNA sequencing, proteomics, lipidomics) allow researchers to analyze multiple molecular layers within individual cells or samples, which provides a more detailed and unbiased understanding of complex biological processes (22, 23). These advancements have allowed for a better understanding of the local and systemic effects of secreted proteins and lipids. Circulating proteins and lipids are important pathogenic elements in both obesity and age-related disease processes; however, large-scale proteomics and lipidomics during over- or undernutrition, especially in the bone marrow compartment, are relatively scarce. Human serum contains a dynamic flux of proteins and lipids that are synthesized by tissues and cells of the body (24). These circulating proteins and lipids regulate global homeostasis via intercellular communication, immune responses, vascular and endothelial cell function, tissue remodeling, fluid exchange, and nutrient assimilation (25). In this study, we used unbiased multi-omics of both human bone marrow and peripheral blood sera to better understand how BMAT and the BM microenvironment respond to HCD and fasting. Our multi-omics study combined transcriptomics of BMAT as well as proteomics and lipidomics of the BM serum and peripheral blood serum to better understand the changes and complex interactions with local and systemic environments.

## 2 Materials and methods

### 2.1 Original study protocol

Fazeli et al., in 2021, studied 23 individuals (10 women and 13 men; mean age: 33.3 years and age range: 22–44 years) of normal weight or overweight (BMI range: 23.3–27.9). No subject had a history of diabetes mellitus or a history of an eating disorder, and none of the participants were taking chronic medications. All women were premenopausal and had a history of regular menstrual cycles. Further baseline characteristics, including racial demographics, peripheral serum hormone parameters, and BMAT (L4 vertebra, femoral diaphysis, and femoral metaphysis), were previously reported (21, 26). All subjects were admitted to the Translational and Clinical Research Center (TCRC) at the Massachusetts General Hospital for 2 inpatient study visits, totaling up to 20 days (21). Participants were initially admitted for a 10-day high-calorie visit, as described previously (21). Based on the participant’s body weight, the Mifflin St Jeor equation was used with an activity factor of 1.3 to calculate their caloric needs for 7% weight gain (4, 5, 21). Participants were permitted to select menu items with a macronutrient content consisting of 45% to 55% carbohydrates, 30% to 40% fat, and no more than 25% protein. After completion of the high-calorie protocol, subjects returned to their normal diet for 13 to 18 days (median: 15 days), referred to as the 2-week stabilization period (21). Subjects were subsequently re-admitted for a second inpatient visit (fasting visit), during which subjects did not consume any calories for 7 to 10 days but were permitted to drink water ad libitum and received a multivitamin containing 400 IU of cholecalciferol daily as well as 20 mEq of potassium chloride (21). Changes in body composition by MRI/DXA, hormonal parameters, and BMAT by 1H-MRS during the high-calorie visit, 2-week stabilization, and fasting visit were previously reported (21).

#### 2.1.1 Peripheral serum

On the first days of each study visit (Baseline: Day 1 and Day 25; “pre”) and the final high-calorie inpatient day (Day 10; “post”) and final fasting day (Day 35; “post”), blood was drawn from each subject (18).

#### 2.1.2 Bone marrow aspiration

Bone marrow aspirates of the posterior iliac crest were performed following standard protocols in a subset of participants (6, 21). Nine subjects (n = 6 men and n = 3 women) underwent bone marrow aspiration at baseline (Day 1; “pre”) and on the final day (Day 10; “post”) of the high-calorie visit, and the remaining 11 subjects (n = 6 men, n = 5 women) (who did not undergo bone marrow aspiration during the high-calorie visit) had bone marrow aspirations at baseline (Day 25; “pre”) and on the final day (Day 35; “post”) of the fasting visit (21).

#### 2.1.3 Bone marrow adipocyte and sera collection

Collected aspirates were placed in collection tubes with 5 mL of PBS, then spun at 377*g* for 8 minutes at 4°C. The components were subdivided into 3 compartments: compartment 1: pellet, which contained the stromal vascular fraction; compartment 2: the fluid between the pellet and the top layer, which we called bone marrow serum because of its appearance; and compartment 3: bone marrow adipocyte, the top layer of floated cells. The top layer of bone marrow adipocytes (compartment 3) was aliquoted and stored at –80°C. As previously reported, the majority of the cells within the top layer were mature adipocytes, but the possibility that a negligible number of stromal vascular cells were also isolated with the bone marrow adipocytes cannot be excluded. A fraction of the bone marrow serum (compartment 2) was also collected and stored at –80°C (21).

#### 2.1.4 Magnetic resonance imaging and ^1^H-MRS for BMAT quantification

As previously described, ^1^H-magnetic resonance spectroscopy (^1^H-MRS) was performed to determine lipid content in the BM (Siemens Trio, 3T, Siemens Medical Systems) (21). For the L4 vertebra, a voxel measuring 15 × 15 × 15 mm (3.4 mL) was placed within the L4 vertebral body. Single-voxel ^1^H-MRS data were acquired using point-resolved spatially localized spectroscopy (PRESS) pulse sequence without water suppression with the following parameters: echo time of 30 ms, repetition time of 3000 ms, 8 acquisitions, 1024 data points, and receiver bandwidth of 2000 Hz. For the femur, a voxel measuring 12 × 12 × 12 mm (1.7 mL) was positioned within the metaphysis at the inter-trochanteric region, and single-voxel ^1^H-MRS using the same non-water-suppressed PRESS pulse sequence was performed. This process was repeated with voxel placement in the mid-diaphysis. Automated procedures for optimization of gradient shimming and transmit and receive gain were used. Fitting of the ^1^H-MRS data was performed using LCModel software (version 6.1-4A) (Stephen Provencher). Data were transferred from the scanner to a Linux workstation, and metabolite quantification was performed using eddy current correction and water scaling. A customized fitting algorithm for BM analysis provided estimates for all lipid signals combined (0.9, 1.3, and 2.3 ppm). LCModel bone marrow lipid estimates were automatically scaled to unsuppressed water peak (4.7 ppm) and expressed as lipid/water ratio (21).

### 2.2 RNA isolation and RNA-sequencing analysis

BM adipocytes were collected from the BM biopsy of the iliac crest, as previously described (21). A subset of samples from the HCD and fasting cohorts (HCD pre n = 6, post n = 6; Fasting: pre n = 6, post n = 6) were shipped to the Vermont Biomedical Research Network (University of Vermont, Burlington, VT) for total RNA extraction and high-throughput RNA-sequencing (RNA-seq) analysis. Of note, these 6 samples per timepoint were remaining from the original study and were still high-quality for RNA analysis. RNA was extracted using a modified MagMAX mirVana RNA extraction protocol with a double clean-up step added to remove excess phenol (Applied Biosystems A27828). Final RNA was protected by adding Ribolock RNase inhibitor (ThermoFisher, cat. no. EO0381, Waltham, MA, USA) to a final concentration of 0.25 U/µL of RNA. RNA was evaluated for quality based on 28S and 18S rRNA using the RNA Picochip (Agilent cat. no. 5067–1513) on the Agilent Bioanalyzer 2100 (Santa Clara, CA). The 260/280 ratio was assessed on a Nanodrop ND1000 (ThermoFisher, Waltham, MA) and quantified using the RNA HS kit using the Quantus Fluorometer (Promega, Madison, WI).

#### 2.2.1 Library Construction, Sequencing, and Enrichment

RNA-seq libraries for Illumina sequencing were constructed using 6 ng of total RNA with the Takara SMARTer Stranded Total RNA-Seq Kit v2 (634412) and evaluated using the Agilent Bioanalyzer 2100 and DNA HS chip. Equal amounts of each library were pooled for sequencing and combined with 10% PhiX control prior to sequencing using HiSeq 1500 with a single read V3 rapid flow cell for a total of 80bp (Illumina Corp, San Diego, CA). The Illumina sequences were trimmed of bases with a Phred quality score < 15 and any contaminating adapters used in cDNA and sequencing library preparation. Only single end reads that survived trimming and were ≥ 60 bases in length were mapped to the human (GRCh37, version 17, Ensembl 72) genome using StringTie (27, 28). Raw reads were normalized to library DNA input and mapped to the Human reference Genome 38 using StringTie (27, 28) at a depth greater than that prescribed by the 2017 ENCODE guidelines for transcriptome sequencing depth (29). Raw counts were imported and analyzed using the edgeR package (30, 31) and gene names were extracted using ballgown (32) in R (version 4.0.2). Counts were adjusted for library size and normalized using the TMM (trimmed mean of M values) method. Counts were retained only if a gene’s counts per million reads (CPM) were ≤ 2 and occurred in at least three samples. To generate gene expression, gene levels were assessed separately in each of the two sequencing lanes run. P-values were determined based on the comparison of the post to pre groups; high-calorie post:pre (high nutritional effect) and fasting post:pre (low nutritional effect). Differentially expressed (DE) genes were determined to have a p-value < 0.05 and a fold change > 2.0 or a fold change < -2.0. PantherDB (33, 34) and STRING (35) (for protein-coding genes) were used to assign biological functions to differentially expressed upregulated and downregulated genes. Heat maps relating to the Gene Set Enrichment Analysis were generated using the web-based analyzer, Morpheus (36), with row clustering and one minus Pearson correlation.

### 2.3 Gene set enrichment analysis

GSEA was performed as described (37). All protein-coding genes (18,719 genes) from the post:pre HCD and fasting data sets (n = 6 per timepoint) were used for enrichment analyses. The selection of protein-coding genes was not based on significance, therefore ensuring the same subset of genes was used in these enrichment analyses. Using the Molecular Signatures Database (MSigDB) (https://www.gsea-msigdb.org/gsea/msigdb), we selected comparisons against the following gene matrixes: H: Hallmark gene set (50 gene sets), C5: Ontology gene sets (16,107 gene sets, which contains biological process (GO: BP), cellular component (GO: CC), and molecular function (GO: MF), and HPO: Human Phenotype Ontology (5,653 gene sets). Significant gene sets (pathways) were determined as having at least 7 genes within the gene set, having an adjusted p-value < 0.05, and having a normalized enrichment score (NES) > 1.50. For HCD, we saw a significant upregulation in the post-HCD in the human gene set Gene Ontology Biological Process (GOBP), specifically GOBP: Type 2 Immune Response and GOBP: Interleukin 5 Production. For fasting, we saw a significant upregulation in gene set Human Phenotype Ontology (HP), specifically HP: Poor Wound Healing, within the Post-Fasting data set. The data from GSEA was shown as enrichment plots (seen in **Supplementary Figures S3**, **S4**). The primary result is the enrichment score (ES), which reflects the degree to which a gene set is overrepresented at the top or bottom of a ranked list of genes (red circle). The leading-edge subset of a gene set is the subset of genes that contribute most to the ES (i.e., the core enrichment) (red arrow). The bottom portion of the plot shows the value of the ranking metric. The ranking metric measures a gene’s correlation with a phenotype. The value of the ranking metric goes from positive to negative as you move down the ranked list. A positive value indicates correlation with the phenotype profile (indicated with a red dotted line and red square). Heat maps of the genes found within the gene sets were generated using the web-based analyzer, Morpheus (36), with row and column clustering and one minus Pearson correlation, which allowed for the visualization of individual samples.

### 2.4 Serum proteomic and lipidomic analysis

Proteomic and lipidomic analyses were performed on previously collected human peripheral blood and bone marrow sera samples (n = 4 for each dietary phase and timepoint) (21). Of note, these samples were the only paired samples remaining from the original study. By using paired samples, we can make direct comparisons between the BMS and PS within a single dietary phase. Samples were collected through laboratory blood draws and posterior iliac crest bone marrow biopsies, as previously described (21). Two 12 µL serum aliquots per condition were provided to the MaineHealth Institute for Research Proteomics and Lipidomics Core Facility for analysis. The individual samples were analyzed to provide technical duplicates, which were then averaged prior to application of group-level statistical analysis. Lipid extracts were dissolved in methanol/dichloromethane for mass spectrometry (MS) analysis, as described previously (38). For proteomic analysis via MS, 3 µL of serum was added directly to the ProteoExtract Digestion Kit (Calbiochem, Darmstadt, Germany). Separation of tryptic peptides was completed using an Ultimate RSLC system 3000 (ThermoFisher/Dionex) nanoscale liquid chromatograph. Lipidomic and proteomic analyses were completed via 5600 TripleTOF mass spectrometer (Sciex, Framingham, MA), and downstream analyses, including t-tests and principal component analyses (PCAs), were completed for both datasets utilizing MarkerView Software (Sciex). PCA analyses were performed with no weighting, Pareto scaling, and supervised principal component analysis. Protein profiling was completed using Sequential Window Acquisition of all Theoretical Spectra (SWATH) using a data-independent method as described previously (39) and was analyzed using a human-specific ion library, which utilizes multiple fragment ion chromatograms for each protein. Lipids were analyzed using a global, bias-free lipid profiling acquisition technique (MS/MSALL), as previously described (38, 40). The measured peak intensities (m/z) of the whole proteomic and lipidomic datasets were used to generate p-values and fold changes based on the comparison of the post to pre groups; HCD post:pre (high nutritional effect) and Fasting post:pre (low nutritional effect). Proteomic interaction analyses were performed in STRING using an interaction score = 0.700 (high confidence) and by removing disconnected nodes from the network (41). Lipidomic enrichment analysis of significant neutral and negative lipids (p < 0.05) was performed with Lipid Ontology (LION) (42).

### 2.5 Statistics and calculations

For RNA-sequencing, generated gene expression levels were used to determine p-values and fold changes based on the comparison of the post to pre (post:pre) groups; post:pre HCD (high nutritional effect) and post:pre fasting (low nutritional effect). For proteomic and lipidomic datasets, measured peak intensities (m/z) were used to generate p-values (using a paired-sample 2-tailed t-test) and fold changes based on the post:pre comparisons for both interventions. A p-value < 0.05 with a false discovery rate (FDR) < 0.20 was considered significant. Volcano plots were generated using GraphPad Prism 10.4.1 software with protein-coding differential expressed (DE) genes [p-value < 0.05 and a fold change > 2.0 (upregulated) or a fold change < -2.0 (downregulated)] or DE proteins [p-value < 0.05 and a fold change > 1.0 (upregulated) or a fold change < -1.0 (downregulated)]. The parameters for DE proteins were altered due to an insufficient number of proteins meeting our initial criteria. Using these altered parameters, less than 100 DE proteins were identified. Fold-change values for ratios < 1.0 were represented as negative reciprocals of the ratios. Negative fold-changes were calculated using the formula (-1/ratio), meaning a protein with a post:pre fold change of 0.75 was calculated as having a fold change of -1.333. The fold change ratio was used to determine the Log_2_(fold change) for the volcano plots (i.e., Log_2_(0.75) = -0.4150).

## 3 Results

### 3.1 Bone marrow adipocytes show unique molecular profiles after an acute high calorie diet and acute fasting

**Figure 1:**
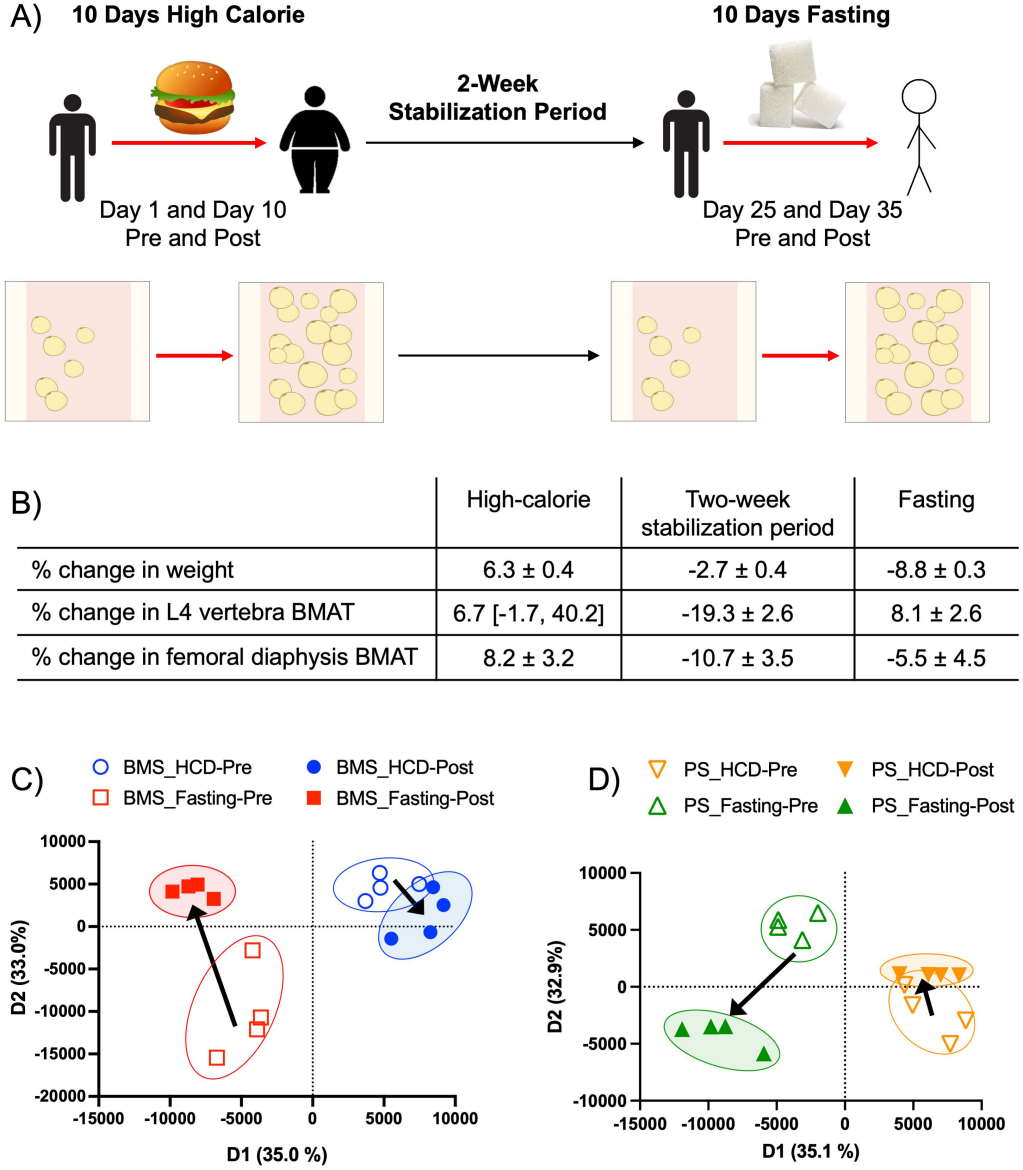
Acute HCD and fasting showed distinct BM adipocyte gene profiles. **(A)** The experimental design of the original patient study. “Pre” and “Post” samples correspond to matching patient samples before then after the dietary intervention. Data were reported as ± SEM or median [IQR] when data were not normally distributed. **(B)** Changes in body and BMAT composition during the HCD, 2-week stabilization period, and fasting intervention. Adapted from Fazeli et al., 2021. **(C)** Proteomic principal component analysis of the BM serum (BMS) after HCD (blue) and fasting (red), illustrating the relationship between samples based on their clustering. Arrows indicate the shift in profiles from the pre (open circle) and post (closed circle) intervention. **(D)** Proteomic PCA of the peripheral blood serum (PS) after HCD (orange) and fasting (green), illustrating the relationship between samples based on their clustering. Arrows indicate the shift in profiles from pre (open circle) to post (closed circle) interventions.

Participants were fed a HCD (caloric needs were calculated for 7% weight gain) for 10 days, then entered a stabilization period of two weeks before starting the 10 days of fasting (**Figure 1A**). As previously reported, participants during the HCD phase had an average change in body weight of 6.3% and an increase in BMAT of 6.7% in the L4 vertebra and 8.2% in the femoral diaphysis (**Figure 1B**) (21). During the stabilization phase, participants returned to their normal diets and lifestyle routine. It was observed that participants had a mean change in body weight of -2.7% (21). During this phase, participants also had a decrease in BMAT of -19.3% in the L4 vertebra and -10.7% in the femoral diaphysis (**Figure 1B**) (21). During the fasting phase, subjects did not consume any calories but received a daily multivitamin containing 400 IU of cholecalciferol and 20 mEq of potassium chloride (21). During this last phase, participants had a change in weight of -8.8%, as well as an increase in BMAT of 8.1% in the L4 vertebra and 5.5% in the femoral metaphysis (**Figure 1B**) (21). The HCD phase, which represented a state of overnutrition, and the fasting phase, which represented a state of undernutrition, both showed significant increases in BMAT. These observations led us to further investigate the molecular and secretory components of BMAT during these two phases. To analyze the effects of acute HCD and acute fasting, we performed RNA-sequencing on isolated BMAT from a subset of participants. Differentially expressed (DE) genes (p-value < 0.05 and fold change (FC) > 2.0 or FC < -2.0) of BM adipocytes showed HCD and fasting resulted in unique gene profiles with minimal overlap in upregulated genes (3 genes or 0.4% overlap) (**Supplementary Figure S1A**). There was also minimal overlap in downregulated genes between the two dietary states (6 genes or 0.1% overlap) (**Supplementary Figure S1B**). None of these overlapping genes are involved in metabolism, immune responses, or inflammatory responses. Interestingly, the fasting phase had 4628 downregulated DE genes while HCD had 45 downregulated DE genes (i.e., a difference greater than 100x) (**Supplementary Figure S1B**). PANTHER analysis of DE genes from BM adipocytes showed the percentage of genes relating to common biological processes; this method allowed us to normalize for the large difference in the number of DE genes between HCD and fasting (505 vs 5000 DE genes). With fasting, there was an increase of ≥ 3.5% in upregulated genes associated with response to stimulus (19.1 vs 15.6%), and metabolic process (30.0 vs 26.2%) (**Supplementary Figure S2A**). Meanwhile, an upregulation of DE genes was found in HCD to be associated with biological regulation (31.1 vs 26.3%) (**Supplementary Figure S2A**). For downregulated DE genes, there was an increase of ≥ 3.5% in genes associated with signaling (13.3 vs 9.2%) in BM adipocytes after fasting compared to the HCD (**Supplementary Figure S2B**). BM adipocytes after HCD showed an increase in downregulated DE genes associated with metabolic processes (34.2 vs 23.5%) compared to fasting (**Supplementary Figure S2B**). When comparing these results, we noticed fasting had more upregulated DE genes associated with metabolic processes, while more metabolic-related DE genes were downregulated with HCD. A PCA of the secreted proteins within the BM serum (BMS), which represents the local microenvironment, and the peripheral blood serum (PS), which represents the systemic environment, allowed us to visualize similarities/differences between our samples through the group clustering, as well as understand their overall relationship on a 2-dimensional plane. The BMS and PS PCAs showed distinct profiles with no overlap that shifted in opposite directions with HCD and fasting (**Figures 1C, D**). Despite the phenotypic similarities between HCD and fasting regarding BMAT expansion, these results demonstrated that BMAT responds on a molecular level to nutritional availability in very distinct ways.

### 3.2 Bone marrow adipocytes had an immunosuppressive phenotype after an acute high calorie diet

**Figure 2:**
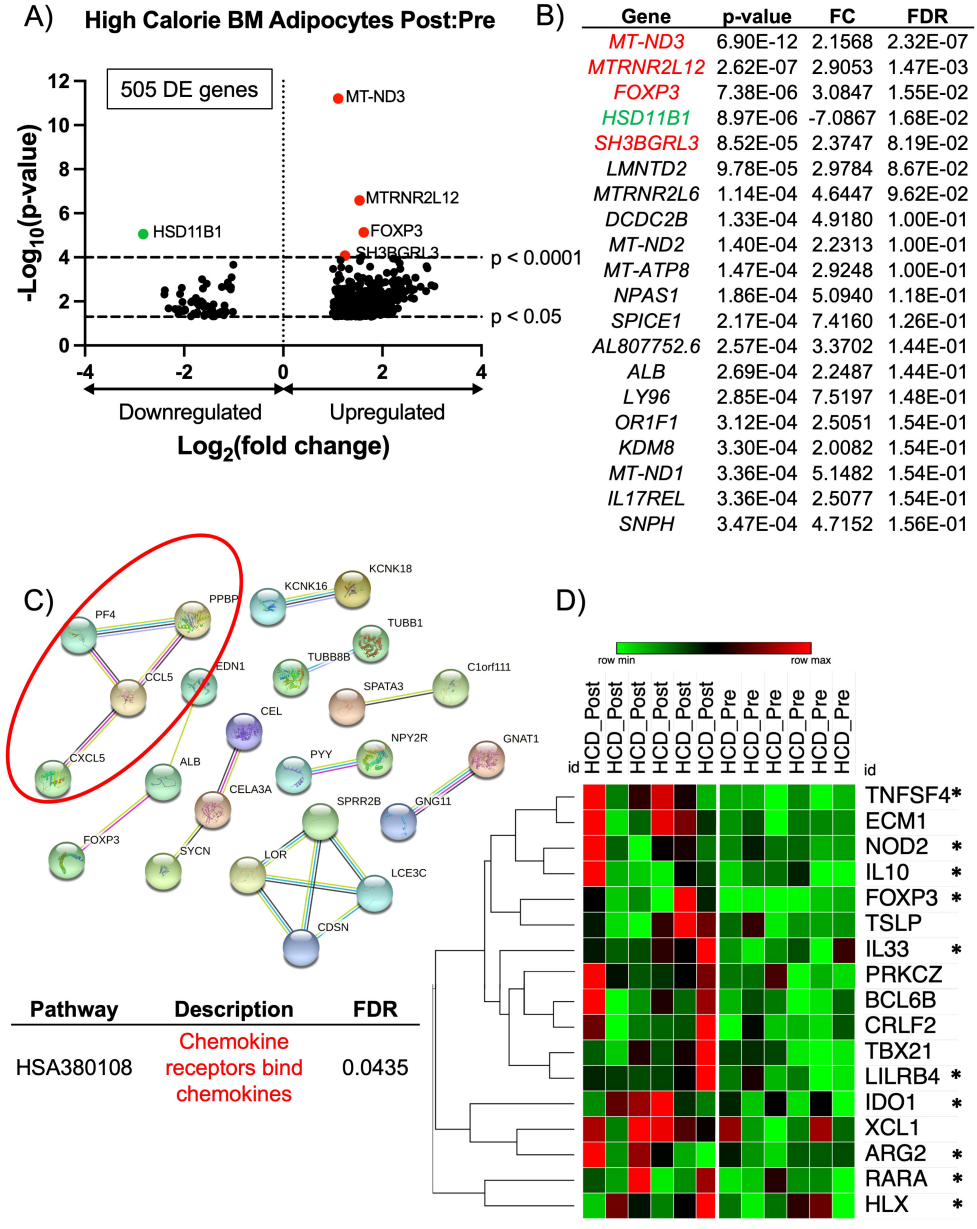
RNA-sequencing analysis of BM adipocytes showed an anti-inflammatory phenotype after high calorie diet. **(A)** Volcano plot of the post:pre comparison after HCD (high nutritional effect) resulted in 505 protein-coding differentially expressed (DE) genes (p-value < 0.05 and FC > 2.0 or FC < -2.0) from BM adipocytes. Genes with a p-value < 0.0001 are above the second dotted line. **(B)** The top 20 genes are listed in order of significance based on p-value. Genes in red are upregulated with a p-value < 0.0001, and genes in green are downregulated with a p-value < 0.0001; these genes correlate to the genes on the volcano plot. **(C)** STRING analysis of the top 193 upregulated protein-coding DE genes based on a FC > 3.0. The network was created using an interaction score = 0.700 (high confidence) and by removing disconnected nodes. **(D)** Heatmap illustrates the individual expressions of genes found in the GSEA (p < 0.05 and normalized enrichment score > 1.50). The asterisks (*) indicate genes with a known anti-inflammatory/immunosuppressive characteristic. Analysis of the heatmap was performed with one minus Pearson correlation and row clustering.

BM adipocytes after the HCD phase had a total of 505 protein-coding DE genes (**Figure 2A**). The four most significantly upregulated DE genes (p < 0.0001) were *MT-ND3*, *MTRNR2L12*, *FOXP3*, and *SH3BGRL3* (indicated in red) (**Figure 2B**). *MTRNR2L12* is a pseudogene, and *MT-ND3* encodes for a subunit of mitochondrial Complex I. The upregulation of *SH3BGRL3* has been linked to cell activation, immune response, and myelopoiesis (43). Like *SH3BGRL3*, *FOXP3* also has immunomodulating properties, which demonstrates that BM adipocytes may have a role in immune cell responses. *FOXP3* functions as a master regulator for the development and function of regulatory T-cells (Tregs) in humans and plays a critical role in maintaining immune tolerance by suppressing immune responses (44, 45). Although *FOXP3* expression has not been previously reported in BM adipocytes, there has been evidence of SSCs that express *FOXP3* during adipogenesis (46). The most significantly downregulated DE gene (p < 0.0001) was *HSD11B1* (indicated in green) (**Figure 2B**). *HSD11B1* regulates cortisol production from cortisone, and its upregulation has been associated with stress, chronic inflammatory conditions like atherosclerosis, inflammatory bowel disease, and colitis (47). Since the majority of HCD DE genes were upregulated, we performed a STRING analysis to determine the degree of co-expression between genes with a FC > 3.0. The STRING analysis showed a significant cluster of genes related to chemokine receptors binding chemokines (*CXCL5*, *CCL5*, *PF4*, and *PPBP*) (**Figure 2C**). Upon further investigation, this cluster of genes has been reported to have immunosuppressive properties. *CXCL5* (encodes for neutrophil-activating peptide 78) promotes immunosuppression by activating myeloid-derived suppressor cells (MDSC), which are inflammatory cells that suppress the immune system (48, 49). *CCL5* (encodes for C-C motif chemokine 5) has been shown to promote immunosuppression in humans and mice by attracting immunosuppressive T-cells and MDSC and exacerbating insulin resistance (50–52). Lastly, *PF4* (encodes for platelet factor 4) can suppress the immune system by reducing cytokine release and inhibiting T-cell function (53). Based on these results, we widened our investigation to include all significantly upregulated genes (p < 0.05), regardless of FC, for a more unbiased analysis. We performed a gene set enrichment analysis (GSEA) and found two significant human gene sets: GOBP: Type 2 Immune Response (p = 0.01365 and NES = 1.697) and GOBP: Interleukin 5 Production (p = 0.00792 and NES = 1.696) (**Supplementary Figures S3A, B**). Of the 17 genes associated with the core enrichment from the GSEA data sets, 10 genes have been reported to have immunosuppressive and/or anti-inflammatory properties (**Figure 2D**). Taken together, and contrary to previous human and rodent studies that illustrate BMAT as inflammatory in obese states (21, 54), our results demonstrate that BM adipocytes have an immunomodulatory/immunosuppressive phenotype after acute HCD.

### 3.3 Acute fasting resulted in an inflammatory molecular phenotype in bone marrow adipocytes

**Figure 3:**
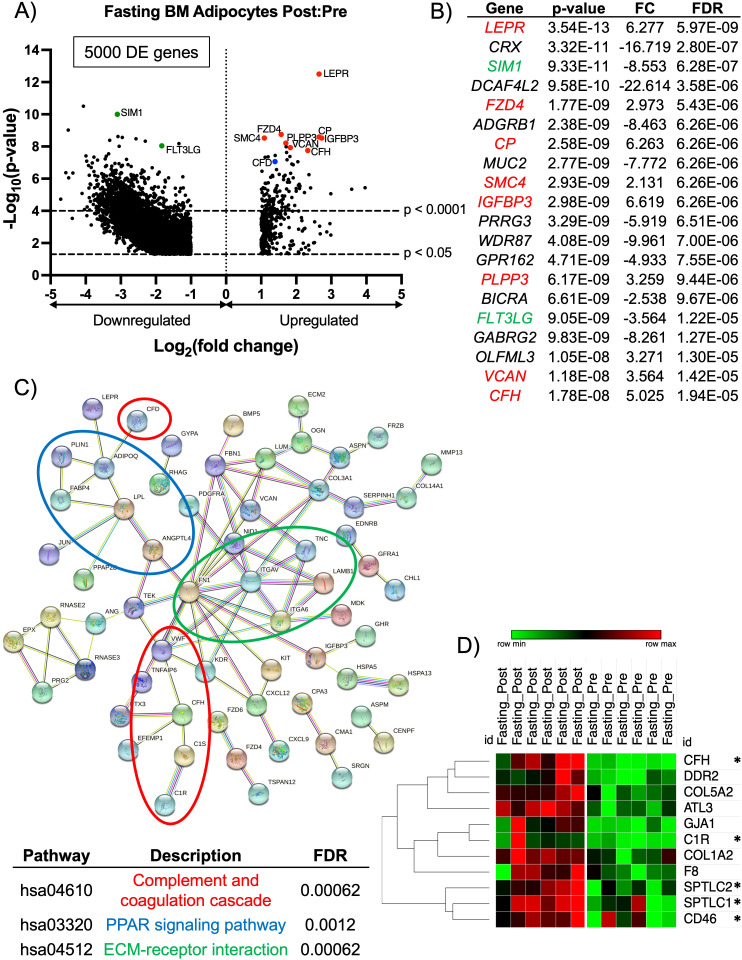
RNA-sequencing analysis of BM adipocytes showed a pro-inflammatory phenotype after fasting. **(A)** Volcano plot of the post:pre comparison after fasting (low nutritional effect) resulted in 5000 protein-coding DE genes (p-value < 0.05 and FC > 2.0 or FC< -2.0) from BM adipocytes. Genes with a p-value < 0.0001 are above the second dotted line. **(B)** The top 20 genes are listed in order of significance based on p-value. Genes in red are upregulated with a p-value < 0.0001, and genes in green are downregulated with a p-value < 0.0001; these genes correlate to the genes on the volcano plot. **(C)** STRING analysis of the top 147 upregulated protein-coding DE genes based on a FC > 2.5. The network was created using an interaction score = 0.700 (high confidence) and by removing disconnected nodes. **(D)** Heatmap illustrates the individual expressions of genes found in the GSEA (p < 0.05 and normalized enrichment score > 1.50). The asterisks (*) indicate genes with a known inflammatory/complement pathway characteristic. Analysis of the heatmap was performed with one minus Pearson correlation and row clustering.

BM adipocytes after the fasting phase had a total of 5000 protein-coding DE genes (**Figure 3A**). The top 20 DE genes, based on p-value, showed 8 upregulated DE genes (*LEPR*, *FZD4*, *CP*, *SMC4*, *IGFBP3*, *PLPP3*, *VCAN*, *CFH*) (indicated in red) and 2 downregulated DE genes (*SIM1* and *FLT3LG*) (indicated in green) were associated the complement pathway and/or a pro-inflammatory response (**Figure 3B**). Another significantly upregulated gene related to the complement pathway was *CFD* (p < 0.0001, FC = 2.632, FDR < 0.0001; indicated by a blue dot). In addition to their inflammatory phenotype, some of the genes have been shown to regulate bone hemostasis. Specifically, *CP* (complement factor C3) has been shown to have a role in osteoclastogenesis, and *IGFBP3* has been shown to upregulate BM adipogenesis through chordin-like 1 stabilization (55, 56). Also, *CFD* (or adipsin) is an adipokine and serine protease that regulates the alternative complement pathway, and is associated with the induction of BM adipogenesis (14). Due to the most significantly upregulated DE genes showing a profile related to the complement pathway and inflammatory responses, we investigated upregulated DE genes based on their FC. Therefore, we performed a STRING analysis to determine the degree of co-expression between upregulated DE genes with a FC > 2.5 in BM adipocytes post-fasting. The STRING analysis showed significant gene clusters related to the complement and coagulation cascade (*CFD*, *VWF*, *TNFAIP6*, *CFH*, *C1S*, *C1R*), PPAR signaling pathway (*PLIN1*, *ADIPOQ*, *FABP4, LPL*, *ANGTL4*), and ECM-receptor interactions (*FN1*, *ITGAV*, *TNC*, *LAMB1*, *ITGA6*) (**Figure 3C**).

The molecular profile of BM adipocytes after acute fasting had a more inflammatory phenotype, unlike what we observed with the HCD. Based on these results, we widened our investigation to include all significantly upregulated genes (p < 0.05) and performed a GSEA. The GSEA found the human gene set, HP: Poor Wound Healing (p = 0.01646 and NES = 1.704), was significantly upregulated post-fasting (**Supplementary Figure S4**). Of the 11 genes associated with the core enrichment from the GSEA data set, 5 genes have been implicated in the complement pathway and immune cell differentiation and activation (**Figure 3D**). Complement factor H (*CFH*) primarily controls the alternative pathway of complement activation, which is crucial for early immune responses against pathogens (57, 58). *C1R* acts as a serine protease that is part of the C1 complex, initiating the classical pathway by activating C1s (59). *SPTLC1* plays a role in immune responses by regulating the differentiation of CD4^+^ T-cells and the development of myeloid cells (60). *SPTLC2* helps maintain T-cell metabolic fitness by translating extracellular signals into intracellular anabolic signals (61). *SPTLC2* has also been described as a ceramide biosynthetic gene that is activated through saturated fatty acids stimulating toll-like receptor 4 (TLR4) (62) Finally, *CD46*, which encodes for membrane cofactor protein, acts as a crucial regulator in the complement pathway by serving as a cofactor for the enzyme Factor I (63, 64). Overall, these results demonstrated a striking difference in the way acute HCD and fasting affected the molecular properties of BM adipocytes. HCD resulted in an immunosuppressive phenotype, while fasting led to changes in immune responses and the complement pathway that resulted in increased inflammation.

### 3.4 After an acute high calorie diet, the bone marrow serum and peripheral serum had an increase in inflammatory markers and regulator proteins for lipid metabolism

**Figure 4:**
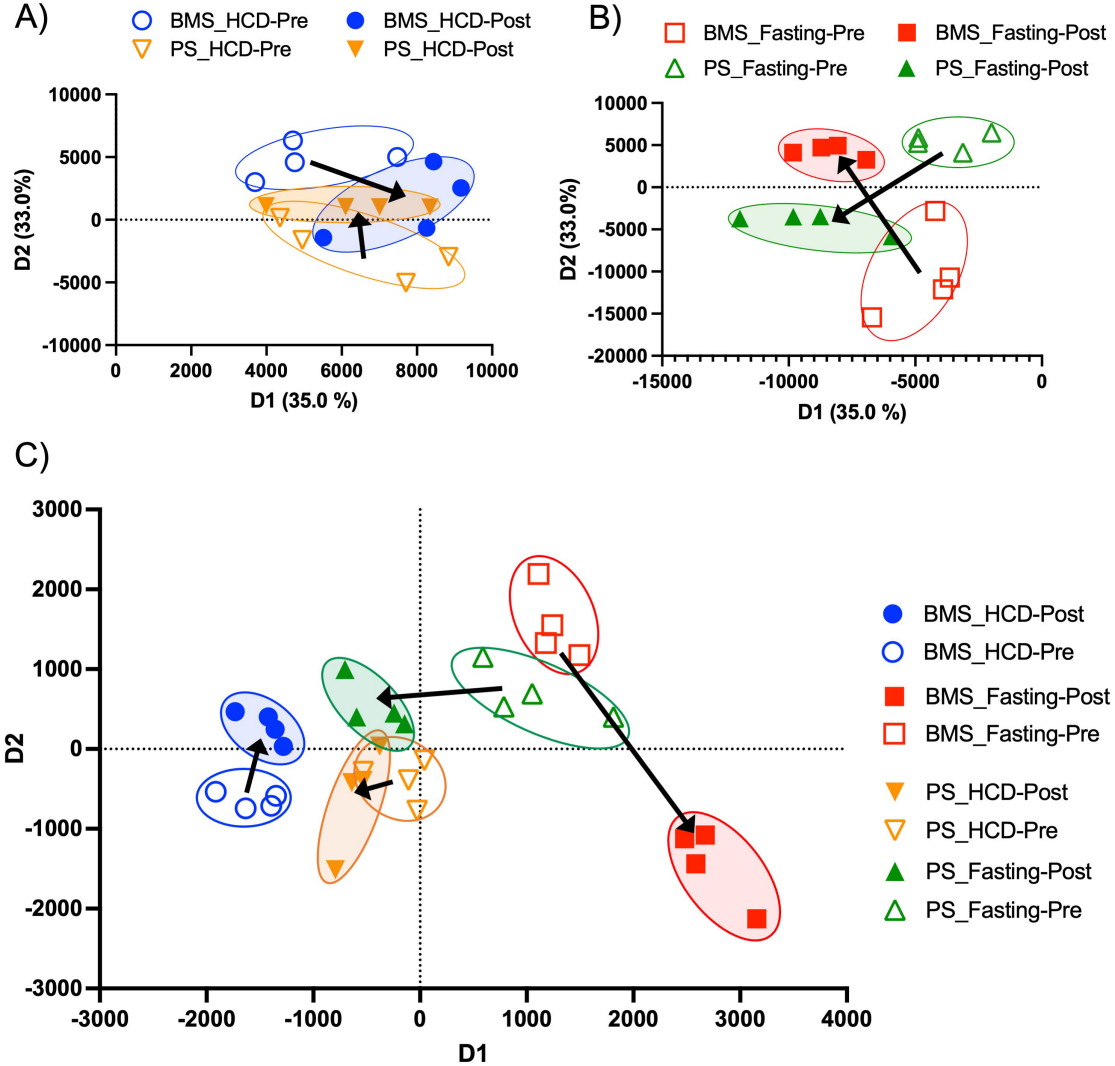
Two-dimensional proteomic and lipidomic PCAs of the BMS and PS after HCD and fasting. **(A)** Two-dimensional proteomic PCA comparing the BMS (blue) and PS (orange) after HCD. Arrows indicate the shift in profiles from the pre (open circle) and post (closed circle) intervention. **(B)** Two-dimensional proteomic PCA comparing the BMS (red) and PS (green) after HCD. Arrows indicate the shift in profiles from the pre (open circle) and post (closed circle) intervention. **(C)** Two-dimensional lipidomic PCA comparing the BMS and PS after HCD and fasting. Arrows indicate the shift in profiles from the pre (open circle) and post (closed circle) intervention.

Since HCD and fasting proved to have differing effects on BM adipocytes, we wanted to see how these changes influenced secreted proteins in the BM microenvironment (local) and periphery (systemic). A PCA showed that the protein profiles of the BMS and PS were more closely related, which may indicate that acute HCD impacts the BMS and PS in a similar manner (**Figure 4A**). Proteomic analysis analyzed the protein levels in peripheral blood serum (PS) and BM serum (BMS) of the HCD and fasting phases. The BMS with HCD had a total of 63 DE proteins (p-value < 0.05 and FC > 1 or FC < -1). RNA sequencing from the BM adipocytes showed an immunosuppressive phenotype, thus, we looked for anti-inflammatory proteins (indicated in blue) and found 3 proteins that have been shown to have anti-inflammatory properties (PRG4, AACT, and ITIH3) (**Supplementary Figures S5A, B**). However, AACT (encoded by *SERPINA1*) and ITIH3 were downregulated in the BMS. To our surprise, we found 5 significantly upregulated proteins (PROC, RBP4, FETUB, KAIN, CFI) related to the complement pathway or pro-inflammatory properties (indicated in red) (**Supplementary Figures S5A, B**). We also found 4 significantly upregulated proteins (APOC3, APOE, APOC2, AFAM) that have been shown to be involved with lipid metabolism (indicated in green) (**Supplementary Figures S5A, B**). Interestingly, Fetuin B (FETUB) can regulate osteogenesis by inhibiting osteoblast mineralization (65). FETUB has also been associated with peripheral insulin resistance in mice and humans (66). When we performed a STRING analysis on the 63 DE proteins, we confirmed that the majority of the proteins are related to complement and coagulation cascade, as well as lipid metabolism (lipase inhibition, lipoprotein clearance, and lipid transport) (**Supplementary Figure S5C**). Compared to the BM adipocytes, the BMS after acute HCD showed a more inflammatory phenotype and alterations to lipid metabolism. Lipid metabolism has been shown to have an essential role in modulating inflammation within the context of acute and chronic diseases (67, 68). Various lipid species are known to possess immunomodulatory and pro- and anti-inflammatory properties, including fatty acids and their metabolites, sterols, complex lipids (e.g., glycerophospholipids and sphingolipids), and lipoproteins (67, 69).

In the PS after HCD, there was a total of 87 DE proteins (**Supplementary Figure S6A**). We observed proteins related to the complement pathway and inflammatory response (PROC, FETUB, VTDB, CFI, KAIN, indicated in red) and lipid metabolism (APOM, APOC3, APOC1, ALBU, indicated in green), which were similar to the proteins identified in the BMS after HCD (**Supplementary Figures S6A, B**). However, in the BMS, all these proteins were upregulated (significantly and non-significantly), whereas in the PS, APOM, ALBU, and VTDB were significantly downregulated (**Supplementary Figures S6A, B**). Apolipoprotein M (APOM) is thought to be negatively related to inflammation. *Apom* ^−/−^ mice fed a high-fat diet were shown to have a higher inflammatory profile in white adipose tissue (WAT), but 50% lower *Apom* gene expression (70). Albumin (ALBU) has been shown to be negatively correlated to increased fat mass in humans, indicating the downregulation of APOM and ALBU in the PS supports an inflammatory phenotype (71). STRING analysis of the 87 DE proteins (upregulated and downregulated) confirmed relationships with the complement and coagulation cascade and lipid metabolism (lipoprotein assembly, remodeling, and clearance) (**Supplementary Figure S6C**). However, we also saw a cluster of proteins related to innate immunity containing the following proteins: ALDOA (FC: -1.408), APOB (FC: 1.313), FETUA (*AHSG*) (FC: -1.077), CRP (FC: -1.572), LBP (FC: -2.088), C4BPA (FC: -1.107) (**Supplementary Figure S6C**). According to our proteomic analysis, the majority of these proteins were downregulated, which is indicative of reduced immune responses from macrophages and neutrophils that can be caused by factors such as aging, stress, or malnutrition (72–75). Upon further investigation, we discovered several of these proteins are commonly downregulated in responses to inflammation. Alpha-2-Heremans-Schmid glycoprotein (AHSG) is an acute-phase protein that is lowered during inflammation (76). Lipopolysaccharide binding protein (LBP) has been shown to be downregulated in response to inflammation, potentially as a mechanism to limit excessive immune response and prevent tissue damage (77, 78). And complement component 4 binding protein alpha (C4BPA) expression tends to decrease with inflammation to inhibit complement-mediated inflammation (79, 80). Therefore, these results indicated that after acute HCD, the PS begins to show signs of an increased inflammatory response that may be due to the rapid increase in body weight and fat mass seen in the participants after 10 days of HCD.

### 3.5 Secreted proteins in the bone marrow serum, but not peripheral serum, showed increased anti-inflammatory markers after acute fasting

In contrast to HCD, the PCA of the BMS and PS showed unique, non-overlapping profiles that shifted in opposing directions at the pre- and post-fasting phase (**Figure 4B**). The BMS after fasting had a total of 93 DE proteins (p-value < 0.05 and FC > 1 and FC < -1) (**Supplementary Figure S7A**). RNA sequencing of BM adipocytes after fasting showed an increase in inflammation through the complement pathway, thus, we wanted to investigate the effect that inflamed BM adipocytes had on the BMS and PS. Interestingly, the BMS had a downregulation of inflammatory/complement pathway proteins (FETUB, FETUA, C1QC, and RBP4, indicated in red) (**Supplementary Figures S7A, B**). We also found several upregulated (APOF and APOM) and downregulated (APOA4, APOH, and APOC1, indicated in green) DE proteins associated with lipid metabolism (**Supplementary Figures S7A, B**). STRING analysis of the 93 DE proteins showed clusters of proteins related the complement activation, response to stress, regulation of insulin growth factors (IGF) transport and IGFBPs, and defense response (**Supplementary Figure S7C**). The proteins related to stress and defense response were downregulated: APOA4 (FC: -5.005), APOA2 (FC: -1.352), APOC1 (FC: -3.176), SAA4 (FC: -1.247), CD44 (FC: -1.342), APOE (FC: -1.342), HBD (FC: -4.164), HBB (FC: -1.997), PRDX2 (FC: -3.542), and HBG2 (FC: -2.842). These proteins regulate the innate immune response and are negative regulators of inflammation (81–84). Within the clusters, the proteins associated with the regulation of IGF transport and IGFBPs, PROC (FC: 1.3565), ZPI (*SERPINA10*) (FC: 1.3647), SPP2 (FC: 1.6723), were upregulated. Studies have shown that increased levels of IGFBP3 can stimulate lipid droplet formation, adipogenesis, and *de novo* lipogenesis (85, 86). Low serum IGF-1 has also been shown as a biochemical marker for malnutrition (87). After fasting, the BMS appeared to have an anti-inflammatory phenotype with evidence of increased lipid accumulation and metabolism.

The PS after fasting had 79 DE proteins (**Supplementary Figure S8A**). The majority of the DE proteins were downregulated, including the top 20 most significant proteins (**Supplementary Figure S8B**). STRING analysis of the 79 DE proteins revealed clusters of the proteins related to the regulation of lipid biosynthesis, as well as the initial and regulation of the complement cascade (**Supplementary Figure S8C**). The proteins associated with the regulation of lipid biosynthesis, APOB (FC: 1.411), APOA1 (FC: -1.2694), and APOA4 (FC: -5.1734), were mostly downregulated. And the proteins related to the complement pathway, C1QB (FC: -1.1972), C1QC (FC: -1.1703), and C1R (FC: -1.4167), were also downregulated. Taken together, the BMS after acute fasting had an inflammatory phenotype, but this inflammatory effect was not seen systemically after 10 days of fasting. The PS, unlike the BMS, showed evidence of decreased lipid biosynthesis.

### 3.6 Lipid signaling was downregulated in the bone marrow serum after high calorie diet, but not after fasting

**Figure 5:**
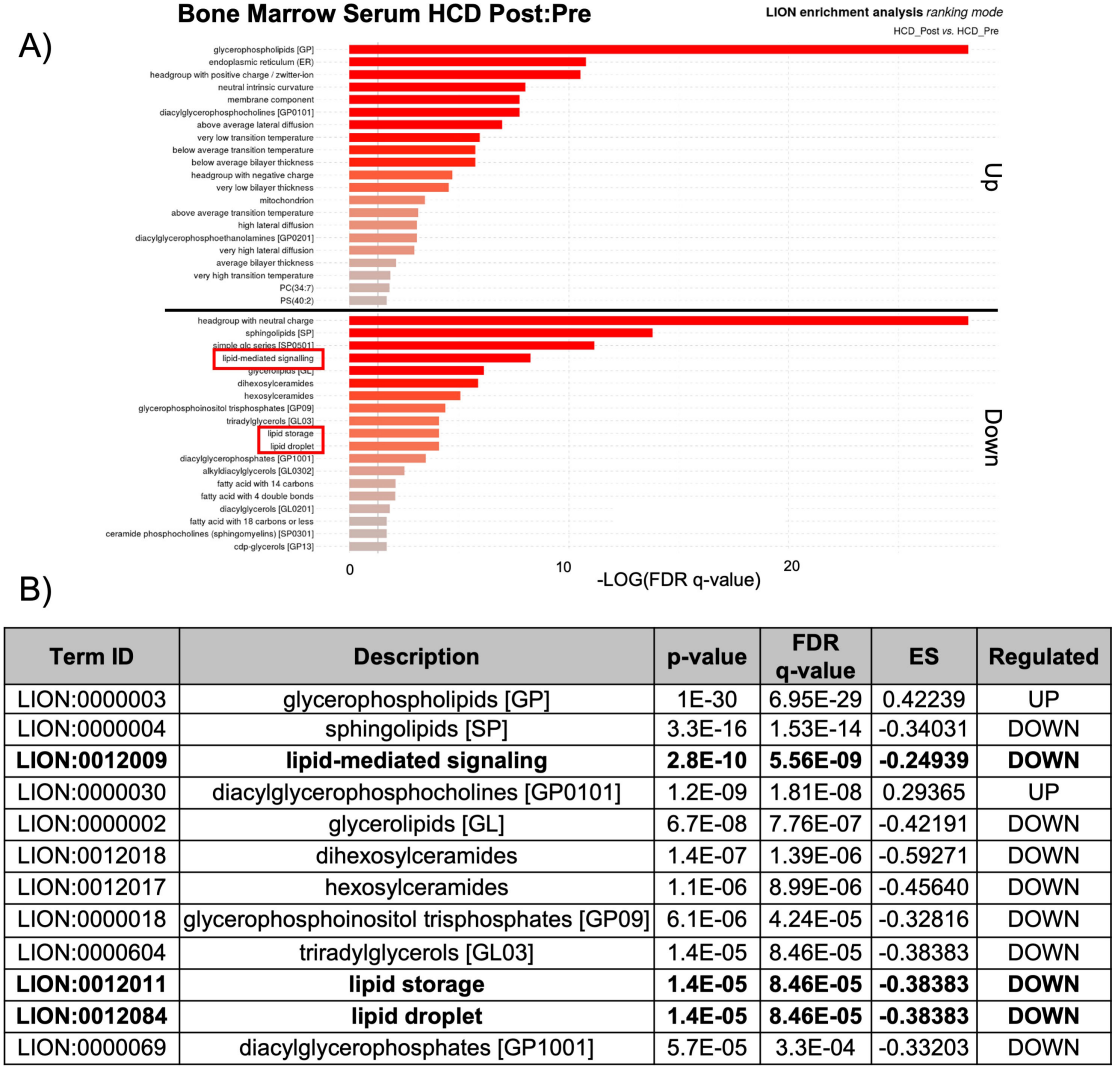
Lipid storage, biogenesis, and signaling were downregulated after HCD. **(A)** Lipid ontology (LION) enrichment analysis of significant positive and negative lipids (p-value < 0.05) from the BMS lipidomic analysis showed lipid-mediated signaling and lipid storage, and lipid droplet biogenesis were significantly downregulated after HCD (outlined in red). **(B)** Top 12 LION terms based on p-value in the BMS after HCD.

**Figure 6:**
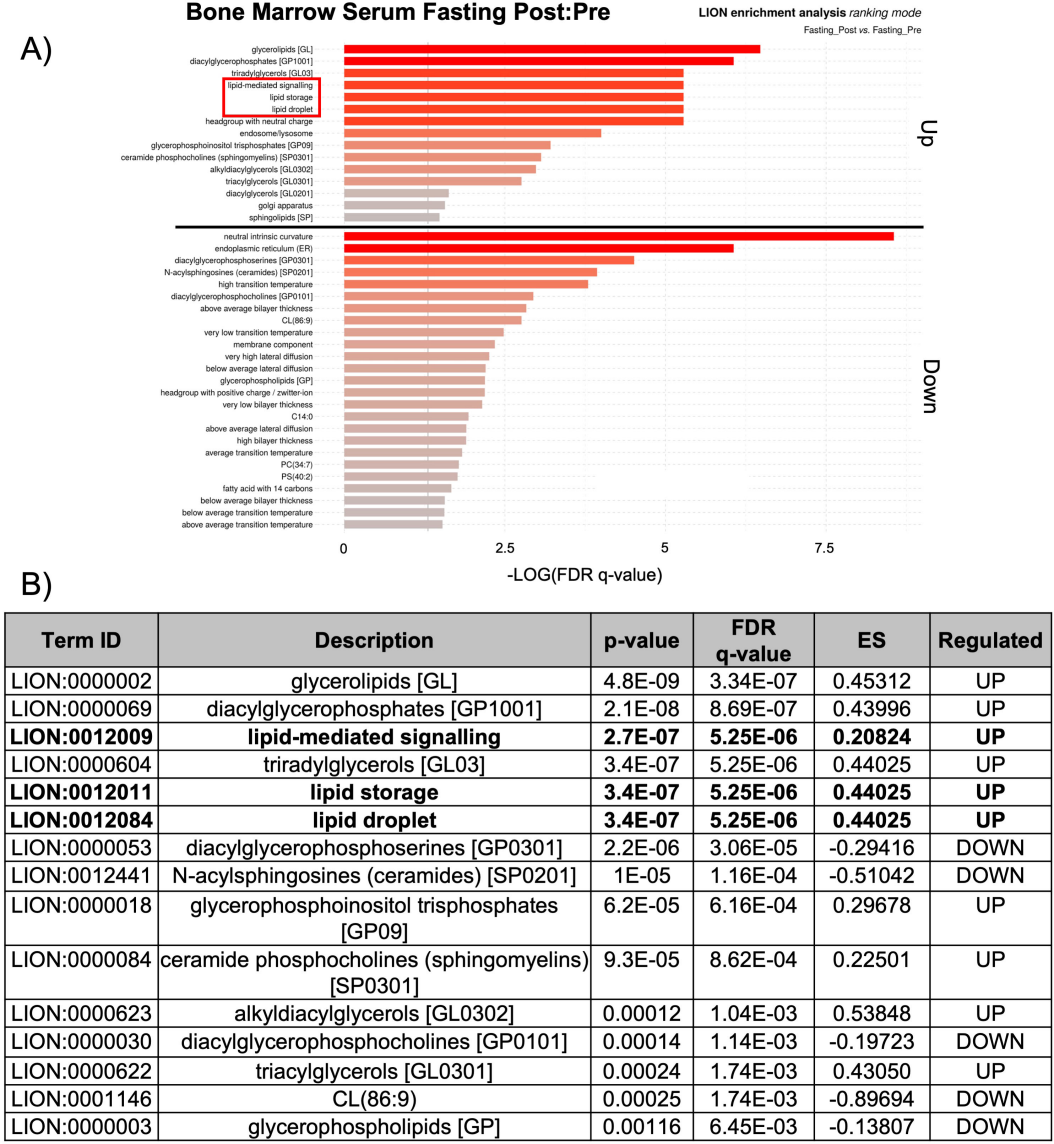
Lipid storage, biogenesis, and signaling were upregulated after fasting. **(A)** Lipid ontology (LION) enrichment analysis of significant positive and negative lipids (p-value < 0.05) from the BMS lipidomic analysis showed lipid-mediated signaling and lipid storage, and lipid droplet biogenesis were significantly downregulated after HCD (outlined in red). **(B)** Top 15 LION terms based on p-value in the BMS after fasting.

**Figure 7:**
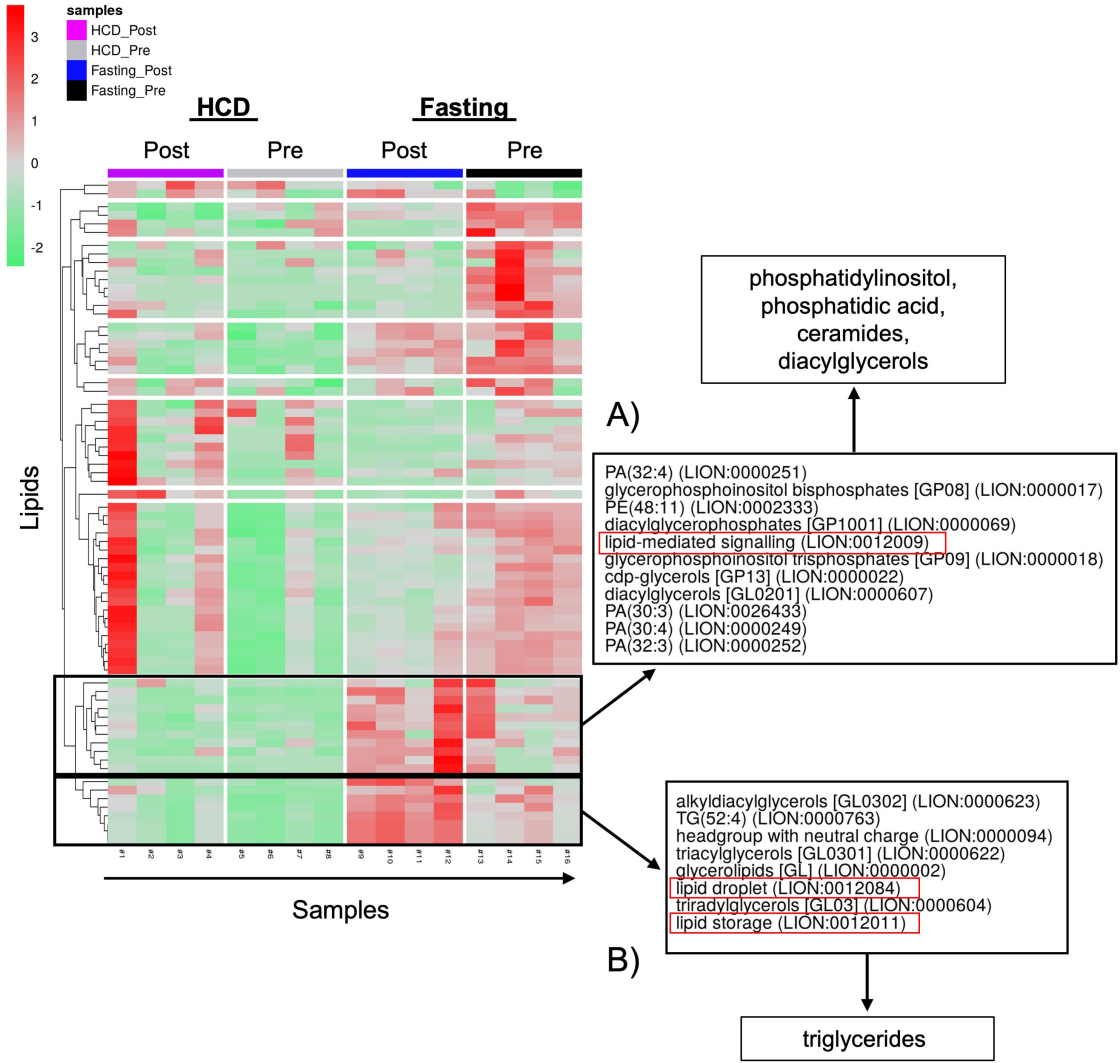
PCA of positive and neutral lipids showed that different classes of lipids are responsible for lipid signaling and lipid storage, and biogenesis. **(A)** The lipids responsible for lipid signaling are phosphatidylinositols (PI), phosphatidic acids (PA), ceramides, and diacylglycerols (DAGs). **(B)** The lipid class responsible for lipid droplet formation and lipid storage is triglycerides (TGs).

The observed changes in proteins related to lipid biosynthesis in the BMS and PS after acute HCD and acute fasting led us to investigate the lipid profiles of the local and systemic environments. A lipidomic PCA of the BMS after HCD and fasting showed that both dietary states resulted in unique, non-overlapping profiles that shifted in opposing directions (**Figure 4C**). The PCA of the PS revealed that the lipid profiles after HCD and fasting had similar shifts in profiles. Lipid ontology (LION) enrichment analysis of the lipids in the BMS after HCD showed a significant downregulation of lipid-mediated signaling (enrichment score: -0.24939) and lipid storage and lipid droplet biogenesis (enrichment score: -0.38383) (**Figures 5A, B**). Within the BMS after HCD, glycerophospholipids and diacyl-glycerophosphocholines were found to be upregulated, while sphingolipids and glycerolipids were downregulated (**Figure 5B**). In the BM, sphingolipids, particularly ceramides, primarily function as signaling molecules regulating cell differentiation, proliferation, and the regulation of hematopoietic progenitor cells (88). In contrast, LION enrichment analysis from BMS after fasting showed lipid storage and lipid droplet biogenesis (enrichment score: 0.44025), and lipid-mediated signaling (enrichment score: 0.20824) were significantly upregulated (**Figures 6A, B**). After fasting significantly, upregulated lipids included glycerolipids (alkyldiacylglycerols and triacylglycerols) and sphingomyelins, while downregulated lipids included N-acylsphingosines and glycerophospholipids (diacylglycerophosphoserines and diacylglycerophosphocholines) (**Figure 6B**). A PCA showed that in post-fasting BMS, there was an increase in the lipids responsible for lipid-mediated signaling: phosphatidylinositol, phosphatidic acid, ceramides, and diacylglycerols (**Figure 7A**, **Supplementary Table S1**). Additionally, triglycerides associated with lipid storage and lipid droplet biogenesis were increased post-fasting compared to post-HCD (**Figure 7B**, **Supplementary Table S1**). These support the finding of the LION enrichment analysis. The PS after HCD and fasting did not show changes in lipid signaling or lipid droplet biogenesis, as observed in the BMS. In the PS after HCD, glycerophospholipids and diacylglycerophosphocholines are upregulated, which is similar to the lipid profile in the BMS after HCD (**Supplementary Figure S9**). After the fasting, the PS showed a similar lipid profile as the BMS, with glycerolipids and sphingomyelins being upregulated (**Supplementary Figure S10**).

## 4 Discussion

This study used an unbiased multi-omics approach, which provided a comprehensive view of biological systems in the BM at multiple molecular levels, to investigate the local and systemic changes that occurred with acute HCD and acute fasting. Previously, we showed that BMAT increased significantly during an acute HCD and fasting intervention, while resulting in a significant increase and decrease in body weight, respectively (21). Consistent with our previous findings, proteomics analysis revealed that the BMS and PS after HCD had an increase in immune response and pro-inflammatory proteins (PROC, RBP4, FETUB, KAIN, and CFI). However, in our current study, we showed that BM adipocytes after HCD had an immunosuppressive/anti-inflammatory molecular phenotype with an upregulation of *FOXP3*, *CXCL5*, *CCL5*, *PF4*, and *PPBP.* After fasting, we observed an inflammatory phenotype within the BM adipocytes. For BM adipocytes, there was an upregulation of DE genes associated with the complement pathway (*CFD*, *VWF*, *TNFAIP6*, *CFH*, *C1S*, and *C1R*), while the BMS showed a significant downregulation of key inflammatory proteins (APOA2, APOC1, APOE, SAA4, and CD44).

*FOXP3* is primarily considered a marker for Tregs, but here we showed that BM adipocytes had a significant upregulation of *FOXP3* expression (p < 0.0001 and FC: 3.0847). Obesity has been strongly linked to decreased T-cell function, meaning the immune system’s ability to fight infections is impaired due to a decline in T-cell activity and effectiveness (89). This decrease in immune cell response has been attributed to the inflammatory expansion of WAT (89–91). However, our data suggest that the expansion of BM adipocytes may have a direct effect on immune cell function that, in the context of HCD or obesity, results in an immunosuppressive phenotype. Previous studies have implied that BM adipocytes can modulate immune cell function through the production of adipokines such as leptin, adiponectin, resistin, and visfatin (92–94). These adipokines can directly interact with immune cells to modulate their function, including regulating T-cell differentiation, macrophage activation, and natural killer cell activity (95, 96). In a diet-induced obesity mouse model, Tencerova et al., 2018 demonstrated that BM adipocytes isolated from high-fat diet fed mice had decreased mRNA levels of inflammatory genes (*Tnfα*, *IL1β*, *Lcn2*), which was in contrast to the WAT (97). These results, in combination with our findings, suggest that BM adipocytes have immunomodulating functions that, in the context of HCD and obesity, have an immunosuppressive effect.

Interestingly, HCD and fasting had opposing phenotypes in the BMAT and BMS (i.e., one is inflammatory while the other is anti-inflammatory). We speculated that the accrual of BMAT acts as an energy reservoir within the BM to maintain bone and hematopoietic homeostasis, which is why the expansion occurs rapidly within 10 days of HCD and fasting. Deletion of *Pnpla2* (encodes for ATGL) in BM adipocytes revealed that BM adipocyte lipolysis was crucial for myelopoiesis and bone homeostasis under conditions of energetic stress, including calorie restriction, irradiation, bone regeneration, and cold exposure (98). HCD creates a state of high nutritional availability through an excess of nutrients, including fats, in the circulation. High levels of circulating fats, especially from a diet high in saturated fats, can lead to increased inflammation by activating immune cells, like macrophages within WAT (68, 99). Adipose tissue expansion releases a “distress” signal that causes macrophages to respond, which releases pro-inflammatory cytokines (100, 101). With injury, the immune system initiates innate immunity, as a first line of defense, followed by adaptive immunity (T-cells and B-cells) (102). However, BMAT is dampening the adaptive immune response, which perpetuates local and peripheral inflammation, as seen in the BMS and PS with HCD. Similar to aging, obesity shows persistent BMAT accrual related to a dampened immune system (75). However, exercise can decrease BMAT, similar to the results we observed during the stabilization period (103, 104). The reduction in BMAT may improve immune and inflammatory responses.

Patients with anorexia nervosa experience chronic inflammation due to extreme and prolonged malnutrition, which has profound and detrimental effects on the immune response (105, 106). However, calorie restriction (i.e., undernutrition, not malnutrition) can improve immune function and reduce inflammation, while decreasing circulating fats (107, 108). In animal studies, calorie restriction significantly increased the lifespan and delayed age-related diseases such as cancer, diabetes, and neurogenerative disorders (107). In our study, we observed inflammatory BMAT after fasting, while the BMS was more anti-inflammatory. In support of our findings, a 10-day zero-calorie fast in relatively healthy individuals showed a similar inflammatory response in WAT, while circulating inflammatory markers trended downward by day 10 (109). The acute fasting phase (i.e., 10 days) was not long enough for participants to enter a true state of malnutrition; we speculate this acute fasting phase was similar to the beneficial effects seen with prolonged intermittent fasting (i.e., 48-72 hour fast) (110, 111). Moreover, in respect to HCD, even though participants lost body weight during the stabilization period, not all of the weight gained during the HCD was lost (i.e., an increase in body weight of 6.3% followed by a decrease of -2.7%). Participants’ body weight was still on average 3.6% higher than their initial starting weight before the HCD, hence, the fasting response may differ from when individuals initiate fasting after a regular diet. Taken together, we believe the BMAT responded to the lack of available nutrients during fasting by creating an inflammatory state, while the BMS was affected by the reduction in peripheral circulating fats, thus creating a more anti-inflammatory phenotype.

Overall, the contrast between the protein profiles of BMS and the gene expression profiles of BM adipocytes during states of nutrient flux represents the dynamic nature of the BM. Proteins from the BMS are the sum of multiple secretory events from BM cells that reflect the primary response to nutrient changes or secondary compensatory processes. Further complicating the interpretation of BMS, the clearance rates of these proteins in this compartment are unknown, nor is it known what the relative proportion of circulating proteins is present in the BMS. DE gene profiles from BM adipocytes, on the other hand, likely reflect the dynamic and rapid response to nutrient stress.

Dietary and endogenous lipids possess anti- and pro-inflammatory properties. Lipid metabolism plays an essential role in modulating inflammation within the context of acute and chronic diseases (88, 112, 113). BMAT expansion after HCD and fasting resulted in opposing lipid profiles. After HCD, the BMS had a decrease in lipid-mediated signaling and lipid storage, and lipid biogenesis. In comparison, after fasting, the BMS had an increase in lipid-mediated signaling, lipid storage, and biogenesis. Based on the dietary phase, changes in circulating carbohydrates may be affecting *de novo* lipogenesis. It has previously been shown that *de novo* lipogenesis is upregulated with calorie restriction (98). During HCD and fasting, the BMAT accrual occurred rapidly, which may have altered the enzymes involved in lipid breakdown. The accumulation of signaling lipids, including eicosanoids, phosphoinositides, sphingolipids, and fatty acids, can alter the cellular biochemical foundation and can modulate cell survival and angiogenesis (112, 114). Sphingolipids are a class of lipids that decreased in the BMS after HCD, but increased after fasting. The decrease in sphingolipids after HCD may create a disruption in lipid signaling pathways within the cell that reduces lipid signaling and storage in BM cells. In contrast, when calories are scarce, like during the fasting phase, the oxidation of saturated fats may be impaired, potentially due to metabolic adaptations and the need for the body to prioritize other energy sources like glucose (115). Studies have shown that impaired oxidation of saturated fat can contribute to the accrual of sphingolipids in tissues (116). Accordingly, therapeutic strategies and nutritional interventions that target lipid metabolism are promising approaches to mitigate inflammation and optimize immune function in obesity, cardiovascular disease, chronic metabolic and inflammatory disorders, autoimmunity, and pathogen defense (67).

There are several limitations to this study. First, as noted previously, the fasting phase of this trial followed the HCD protocol for all participants. Therefore, the changes we observed during fasting may have been influenced by changes during the acute HCD that did not return to a homeostatic balance in the two-week interval. Second, our results represent the acute effects of these dietary alterations, which may not be applicable to long-term, chronic diseases such as obesity, type II diabetes mellitus, or anorexia nervosa. Similarly, short-term fasting does not equate with long-term calorie restriction and its impact on metabolic homeostasis. In addition, the samples analyzed were what remained from the original sampling; thus, the sample sizes were small. The BMS and PS for each dietary phase were paired, meaning the samples were from the same participants, and we were able to make direct comparisons between the local and systemic sera. However, this unbiased approach to analyze the local and systemic effects after acute dietary interventions needs to be repeated in a larger study group to confirm our findings. Next, the gene expression studies were performed on floated bone marrow adipocytes; we did not sort those cells, so it is conceivable that some of the cells were macrophages and/or fibroblasts. However, based on the BMAT content found within the iliac crest biopsy, we believe the contamination of other cells is minimal compared to the adipocyte content. Finally, we did not examine other BM cells found within the cell pellet after BM aspirate centrifugation. Analysis of these cells could have provided additional data about the BMS, including alternative sources for the proteins and lipids found in our analysis.

In conclusion, we demonstrated that after an acute 10-day HCD followed by a 10-day fasting protocol that BMAT increased in response to both interventions, but the omics and cues for these changes were drastically different. Under these dietary interventions, BMAT accrual occurred as an energy reservoir thought to maintain bone and hematopoietic homeostasis. However, the local and systemic effects of BMAT expansion proved to be dependent upon nutrient availability. Nevertheless, these results demonstrate changes to anti- and pro-inflammatory markers, immune response markers, and lipid metabolism after HCD and fasting that will be used in future studies to further our understanding of BM adipocytes.

## Data Availability

The RNA-sequencing datasets generated for this study can be found in the NCBI's Sequence Read Archive (SRA) database under the BioProject accession: PRJNA1266176 and in the Gene Expression Omnibus (GEO) under the accession: GSE297963. The mass spectrometry proteomics data have been deposited to the ProteomeXchange consortium via the PRIDE partner repository with the dataset identifier: PXD063552. The lipidomics data have been uploaded to the NIH Common Fund's National Metabolomics Data Repository (NMDR) website, the Metabolomics Workbench, (http://www.metabolomicsworkbench.com) where it has been assigned Study ID: ST003913.The data can be accessed directly via its Project DOI: http://dx.doi.org/10.21228/M86R8C.

## Supplementary material

Supplementary Figure 1 There was minimal overlap in upregulated and downregulated BM adipocyte genes after HCD and fasting. **(A)** Venn-diagram of upregulated DE genes showed that 3 genes were upregulated after both HCD and fasting. **(B)** Venn-diagram of downregulated DE genes showed that 6 genes were downregulated after both the HCD and fasting. The overlapping genes were not associated with inflammatory responses, immune responses, or metabolism.

Supplementary Figure 2 Percentage of upregulated and downregulated DE genes found in common biological processes after HCD and fasting. **(A)** PANTHER analysis of the upregulated DE genes showed the percentage of genes relating to common biological processes. The asterisks (*) highlight biological processes with a ≥ 3.5% difference between HCD and fasting. **(B)** PANTHER analysis of the downregulated DE genes showed the percentage of genes relating to common biological processes. The asterisk (*) highlights biological processes with a ≥ 3.5% difference between HCD and fasting.

Supplementary Figure 3 Gene set enrichment analysis (GSEA) of upregulated genes post-HCD. **(A)** GSEA showed a significant (p-value < 0.05 and normalized enrichment score (NES) > 1.50) upregulation in the post:pre HCD for genes associated with the gene set Gene Ontology Biological Process (GOBP) Type 2 Immune Response. **(B)** GSEA showed a significant (p-value < 0.05 and normalized enrichment score > 1.50) upregulation in the post:pre HCD for genes associated with the gene set GOBP Interleukin 5 Production. The primary result is the enrichment score (ES), which reflects the degree to which a gene set is overrepresented at the top or bottom of a ranked list of genes (red circle). The leading-edge subset of a gene set is the subset of genes that contribute most to the ES (i.e., the core enrichment) (red arrow). The bottom portion of the plot shows the value of the ranking metric. The ranking metric measures a gene’s correlation with a phenotype. The value of the ranking metric goes from positive to negative as you move down the ranked list. A positive value indicates correlation with the phenotype profile (indicated with a red dotted line and red square).

Supplementary Figure 4 Gene set enrichment analysis (GSEA) of upregulated genes post-fasting. **(A)** GSEA showed a significant (p-value < 0.05 and NES > 1.50) upregulation in the post: pre fasting for genes associated with the gene set Human Phenotype Ontology (HP) Poor Wound Healing. The primary result is the enrichment score (ES), which reflects the degree to which a gene set is overrepresented at the top or bottom of a ranked list of genes (red circle). The leading-edge subset of a gene set is the subset of genes that contribute most to the ES (i.e., the core enrichment) (red arrow). The bottom portion of the plot shows the value of the ranking metric. The ranking metric measures a gene’s correlation with a phenotype. The value of the ranking metric goes from positive to negative as you move down the ranked list. A positive value indicates correlation with the phenotype profile (indicated with a red dotted line and red square).

Supplementary Figure 5 Proteomic analysis of the BMS after HCD had a pro-inflammatory phenotype. **(A)** Volcano plot of the post:pre comparison after HCD (high nutritional effect) resulted in 63 DE proteins (p-value < 0.05 and FC > 1.0 or FC< -1.0) within the BMS. Proteins with a p-value < 0.0001 are above the second dotted line. **(B)** Top 20 proteins are listed in order of significance based on p-value. Proteins in blue represent are associated with anti-inflammatory properties, proteins in red are associated with inflammatory properties, and proteins in green are associated with metabolism; these proteins correlate to the genes on the volcano plot. **(C)** STRING analysis of 63 DE proteins. The network was created using an interaction score = 0.700 (high confidence) and by removing disconnected nodes.

Supplementary Figure 6 Proteomic analysis of the PS after HCD showed a mild inflammatory phenotype. **(A)** Volcano plot of the post:pre comparison after HCD (high nutritional effect) resulted in 87 DE proteins (p-value < 0.05 and FC > 1.0 or FC< -1.0) within the PS. Proteins with a p-value < 0.0001 are above the second dotted line. **(B)** Top 20 proteins are listed in order of significance based on p-value. Proteins in red are associated with inflammatory properties, and proteins in green are associated with metabolism; these proteins correlate to the genes on the volcano plot. **(C)** STRING analysis of 87 DE proteins. The network was created using an interaction score = 0.700 (high confidence) and by removing disconnected nodes.

Supplementary Figure 7 Proteomic analysis of the BMS after fasting showed an anti-inflammatory phenotype. **(A)** Volcano plot of the post:pre comparison after fasting (low nutritional effect) resulted in 93 DE proteins (p-value < 0.05 and FC > 1.0 or FC< -1.0) within the BMS. Proteins with a p-value < 0.0001 are above the second dotted line. **(B)** Top 20 proteins are listed in order of significance based on p-value. Proteins in red are associated with inflammatory properties, and proteins in green are associated with metabolism; these proteins correlate to the genes on the volcano plot. **(C)** STRING analysis of 93 DE proteins. The network was created using an interaction score = 0.700 (high confidence) and by removing disconnected nodes.

Supplementary Figure 8 Proteomic analysis of the PS after fasting showed a mild inflammatory phenotype. **(A)** Volcano plot of the post:pre comparison after fasting (low nutritional effect) resulted in 79 DE proteins (p-value < 0.05 and FC > 1.0 or FC< -1.0) within the PS. Proteins with a p-value < 0.0001 are above the second dotted line. **(B)** Top 20 proteins are listed in order of significance based on p-value. Proteins in red are associated with inflammatory properties, and proteins in green are associated with metabolism; these proteins correlate to the genes on the volcano plot. **(C)** STRING analysis of 79 DE proteins. The network was created using an interaction score = 0.700 (high confidence) and by removing disconnected nodes.

Supplementary Figure 9 LION enrichment analysis of the PS after HCD. **(A)** Lipid ontology (LION) enrichment analysis of positive and negative lipids (p-value < 0.05) from the PS lipidomic analysis after HCD **(B)**. Top 9 LION terms based on p-value in the PS after HCD.

Supplementary Figure 10 LION enrichment analysis of the PS after fasting. **(A)** Lipid ontology (LION) enrichment analysis of positive and negative lipids (p-value < 0.05) from the PS lipidomic analysis after fasting. **(B)** Top 13 LION terms based on p-value in the PS after fasting.
