# Supplementary material for: Altered metabolomics and inflammatory transcriptomics in human bone marrow adipocytes after acute high calorie diet and acute fasting
Source: Front Endocrinol (Lausanne). 2025 Jun 16;16:1591280. doi: 10.3389/fendo.2025.1591280 (PMC12206641; doi:10.3389/fendo.2025.1591280)
Supplement: Supplementary file 1 [file Table1.pdf]

Supplementary Table 1: The identified lipids that are associated with lipid-mediated signaling, lipid droplet formation, and lipid storage.

Term ID	# of lipids	Identified Lipids
**lipid-mediated signaling (LION:0012009)**	242	PIP3(22:8); PIP3(22:7); PIP3(30:5); PIP(26:2); PIP3(30:0); PIP3(32:0); PIP(32:2); PIP(42:4); PIP3(34:4); PIP3(34:2); PA(26:3); PIP2(32:0); PIP3(30:0); PIP(42:3); PIP3(34:4); PIP3(34:2); PIP2(40:3); PIP3(38:3); PA(28:3); PA(30:3); PA(30:2); PIP(26:5); PIP(28:5); PIP2(22:5); PIP2(24:8); PIP(32:10); PIP2(36:4); PIP2(44:7); PIP3(40:2); PA(28:5); PA(28:4); PA(28:2); PA(44:6); PIP(28:8); PIP(30:8); PIP(30:8); PIP3(34:2); PIP3(34:1); PIP3(36:2); PIP2(44:7); PIP2(44:4); PIP3(38:1); PIP3(38:1); PA(28:6); PIP3(42:6); PIP3(42:5); PIP3(42:2); PA(30:4); PA(30:3); PA(32:4); PA(32:3); PA(36:2); PIP(30:7); PIP2(36:7); PIP2(36:0); PIP3(34:0); PIP2(40:1); PIP3(38:7); PIP3(40:12); PIP3(42:9); PIP2(48:8); PA(30:3); PIP(32:10); PIP3(38:12); PIP3(38:0); PIP3(42:11); PIP3(42:3); PA(32:3); PIP2(46:4); PIP(30:10); PIP(32:10); PIP(32:10); PIP2(44:3); PIP2(26:5); PA(28:4); PIP2(40:1); PIP2(40:1); PIP2(42:0); PIP(26:5); PIP(28:6); PIP3(38:7); PIP3(34:4); PIP3(34:2); PIP2(40:3); PIP2(44:4); PIP3(42:8); PA(30:4); PIP(32:7); PIP2(36:2); PA(28:3); PIP3(42:1); PA(32:5); PA(32:3); PA(34:4); PA(44:6); PIP(28:7); PIP2(36:4); PA(32:2); PIP(30:9); PIP3(42:2); PIP2(24:6); PIP2(26:0); PA(26:3); PA(38:2); PA(28:3); PIP2(40:2); PA(30:4); PA(26:2); PIP3(40:2); PA(32:3); PIP(26:6); PIP2(44:4); PA(32:4); PIP(28:6); PA(28:4); PIP(32:7); PIP(32:6); PIP3(38:6); PIP2(48:7); PIP3(36:0); PA(30:2); PA(34:3); PA(46:8); PIP(32:5); PIP(32:9); PIP(30:8); PIP3(42:4); PIP(32:10); PIP2(22:8); PIP2(22:1); PIP2(24:5); PIP2(44:6); PIP3(38:8); PIP3(38:6); PIP2(40:1); PIP2(42:2); PIP(30:7); PIP(28:6); PIP3(24:7); PIP3(26:4); PIP3(28:5); PIP3(32:5); PIP3(32:3); PIP3(38:1); PIP3(40:1); PIP(26:1); PIP(30:5); PIP(28:7); PIP3(38:7); PIP3(40:12); PIP(30:9); PIP3(40:6); PA(32:4); PA(38:2); PIP(30:10); PIP3(44:6); PIP(30:7); PA(34:3); PIP3(36:6); PIP3(44:6); PA(30:8); PA(30:7); PA(32:4); PA(34:10); PA(40:2); PIP(30:5); PIP3(36:5); PIP3(42:0); PIP3(38:7); PIP3(44:3); PA(44:3); PIP3(46:5); PA(32:3); PIP2(40:0); PIP3(42:2); PA(30:4); PIP(32:3); PIP(36:7); PIP(36:5); PIP3(42:11); PIP(36:3); PA(32:2); PIP(26:0); PIP2(22:7); PIP(32:6); PA(44:5); PIP3(30:3); PIP(28:8); PIP3(30:4); PIP3(20:2); PIP(30:9); PIP(30:3); PIP(32:8); PIP2(36:6); PIP(30:7); PIP2(36:5); PIP(32:8); PIP2(22:6); PIP2(24:7); PIP(30:6); PA(44:12); PIP(30:0); PA(42:6); PIP2(22:0); PIP(28:4); PIP2(22:0); PIP(28:0); PIP(26:1); PIP2(22:2); Cer(34:1;O2); Cer(40:1;O2);

		Cer(42:1;O2); Cer(38:1;O3); Cer(42:0;O4); DG(38:1); DG(36:3); DG(36:0); DG(36:0); Cer(42:1;O3); DG(36:2); Cer(32:4;O4); Cer(42:0;O4); Cer(32:4;O4); Cer(32:3;O4); Cer(32:3;O4); Cer(42:1;O3); DG(36:3); DG(40:5); Cer(34:1;O2); Cer(32:4;O2); Cer(32:3;O3); Cer(38:0;O3); Cer(44:4;O3); DG(40:0); DG(32:0); Cer(32:3;O4); Cer(42:1;O4); Cer(42:1;O4); Cer(32:2;O4); Cer(48:3;O4); Cer(34:1;O2); Cer(42:0;O2)
**lipid droplet (LION:0012084 and lipid storage (LION:0012011)**	42	TG(56:1); TG(42:0); TG(O-54:0); TG(O-56:12); TG(56:6); TG(52:4); TG(O-56:2); TG(O-58:3); TG(50:4); TG(O-52:10); TG(52:2); TG(54:2); TG(O-58:1); TG(44:0); TG(54:0); TG(O-56:6); TG(O-56:0); TG(38:0); TG(O-56:0); TG(O-56:1); TG(46:4); TG(52:4); TG(O-56:0); TG(54:6); TG(52:4); TG(O-54:10); TG(54:5); TG(54:4); TG(54:3); TG(O-56:1); TG(O-58:2); TG(52:2); TG(54:5); TG(54:4); TG(O-56:1); TG(O-56:0); TG(56:9); TG(54:2); TG(48:3); TG(50:7); TG(O-52:6); TG(54:2)
