# Supplementary figures and images for: Altered metabolomics and inflammatory transcriptomics in human bone marrow adipocytes after acute high calorie diet and acute fasting
Source: Front Endocrinol (Lausanne). 2025 Jun 16;16:1591280. doi: 10.3389/fendo.2025.1591280 (PMC12206641; doi:10.3389/fendo.2025.1591280)

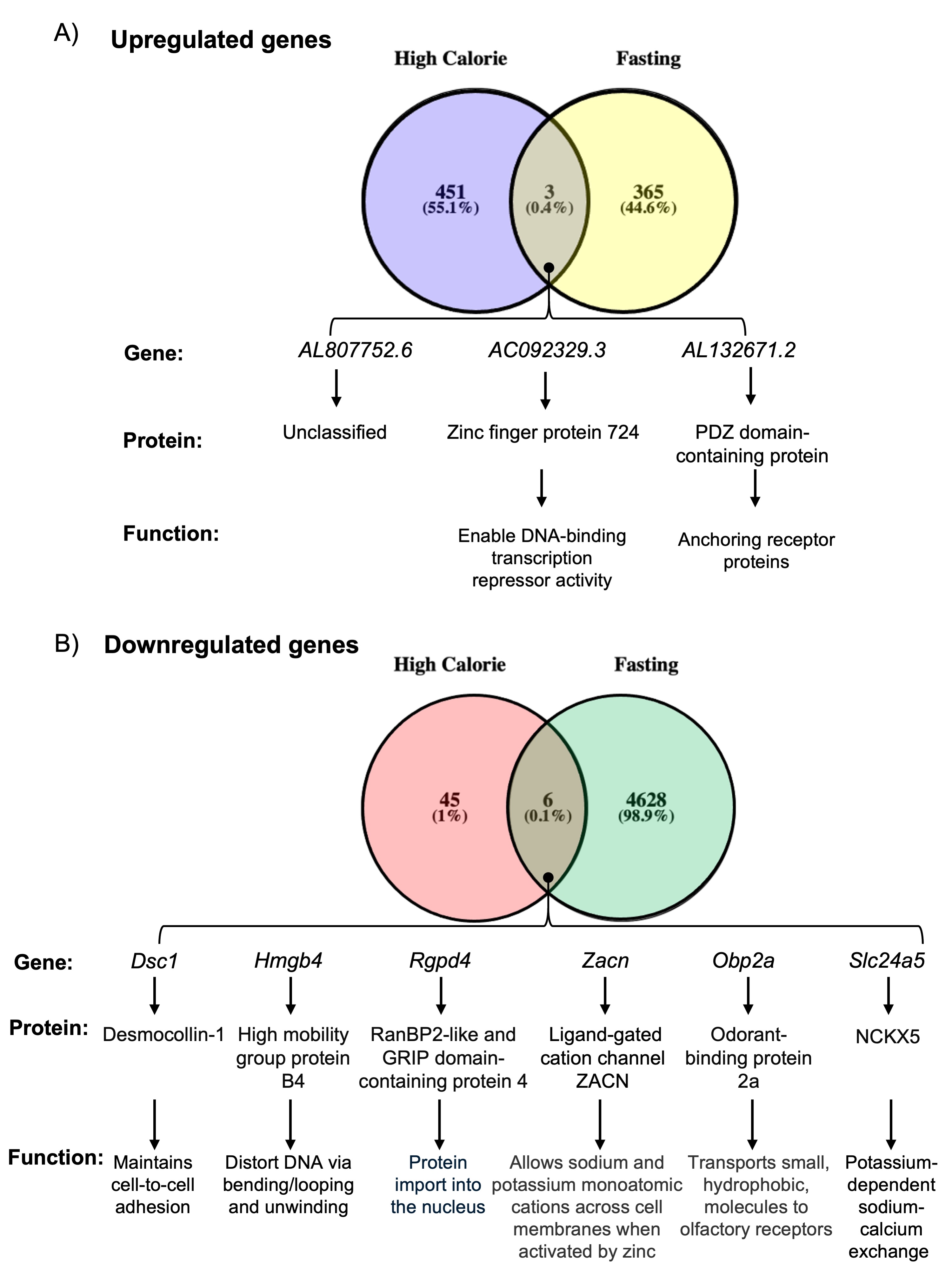

Supplement: Supplementary Figure 1 — There was minimal overlap in upregulated and downregulated BM adipocyte genes after HCD and fasting. (A) Venn-diagram of upregulated DE genes showed that 3 genes were upregulated after both HCD and fasting. (B) Venn-diagram of downregulated DE genes showed that 6 genes were downregulated after both the HCD and fasting. The overlapping genes were not associated with inflammatory responses, immune responses, or metabolism. [file Image1.jpeg]

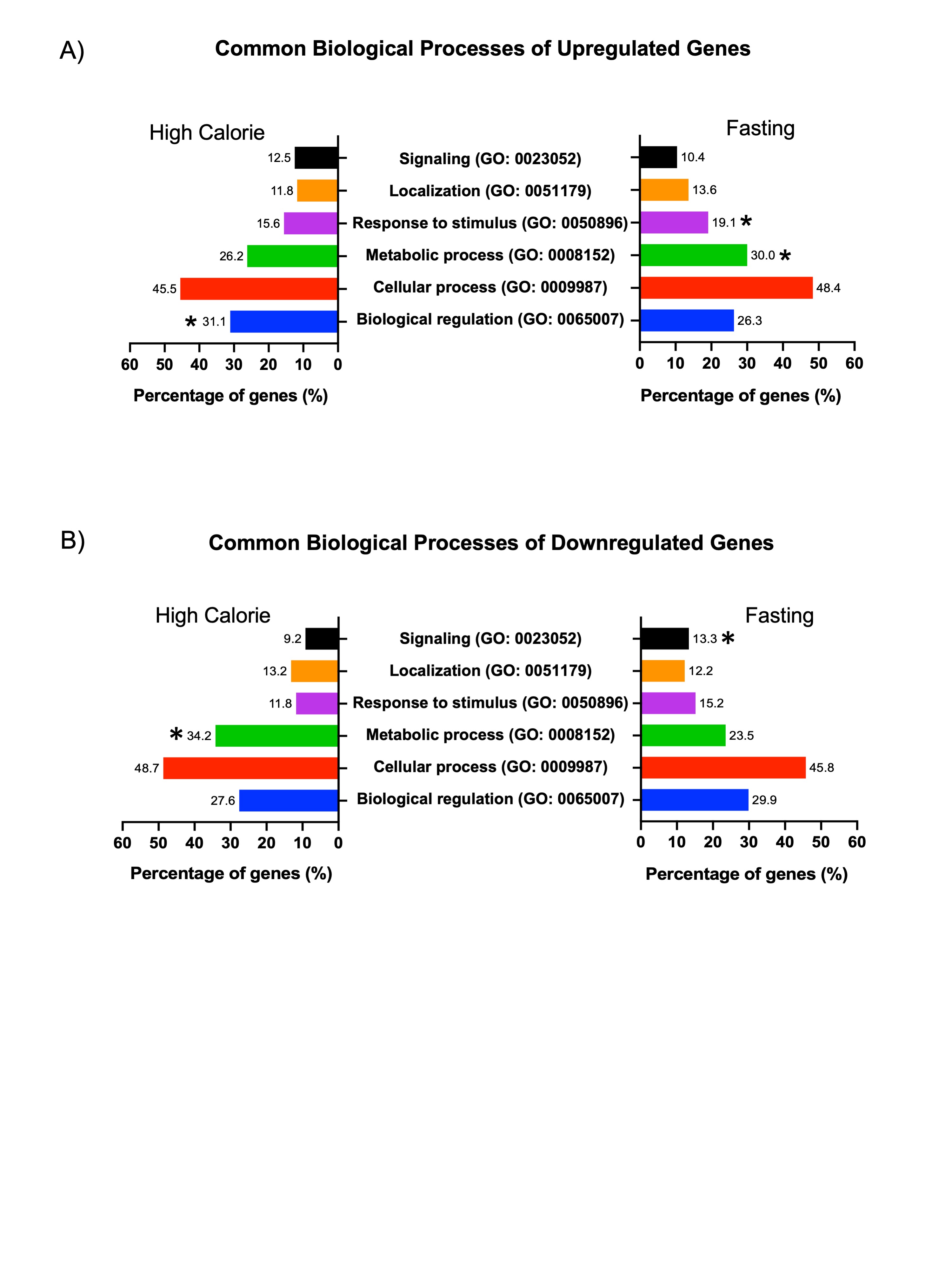

Supplement: Supplementary Figure 2 — Percentage of upregulated and downregulated DE genes found in common biological processes after HCD and fasting. (A) PANTHER analysis of the upregulated DE genes showed the percentage of genes relating to common biological processes. The asterisks (*) highlight biological processes with a ≥ 3.5% difference between HCD and fasting. (B) PANTHER analysis of the downregulated DE genes showed the percentage of genes relating to common biological processes. The asterisk (*) highlights biological processes with a ≥ 3.5% difference between HCD and fasting. [file Image2.jpeg]

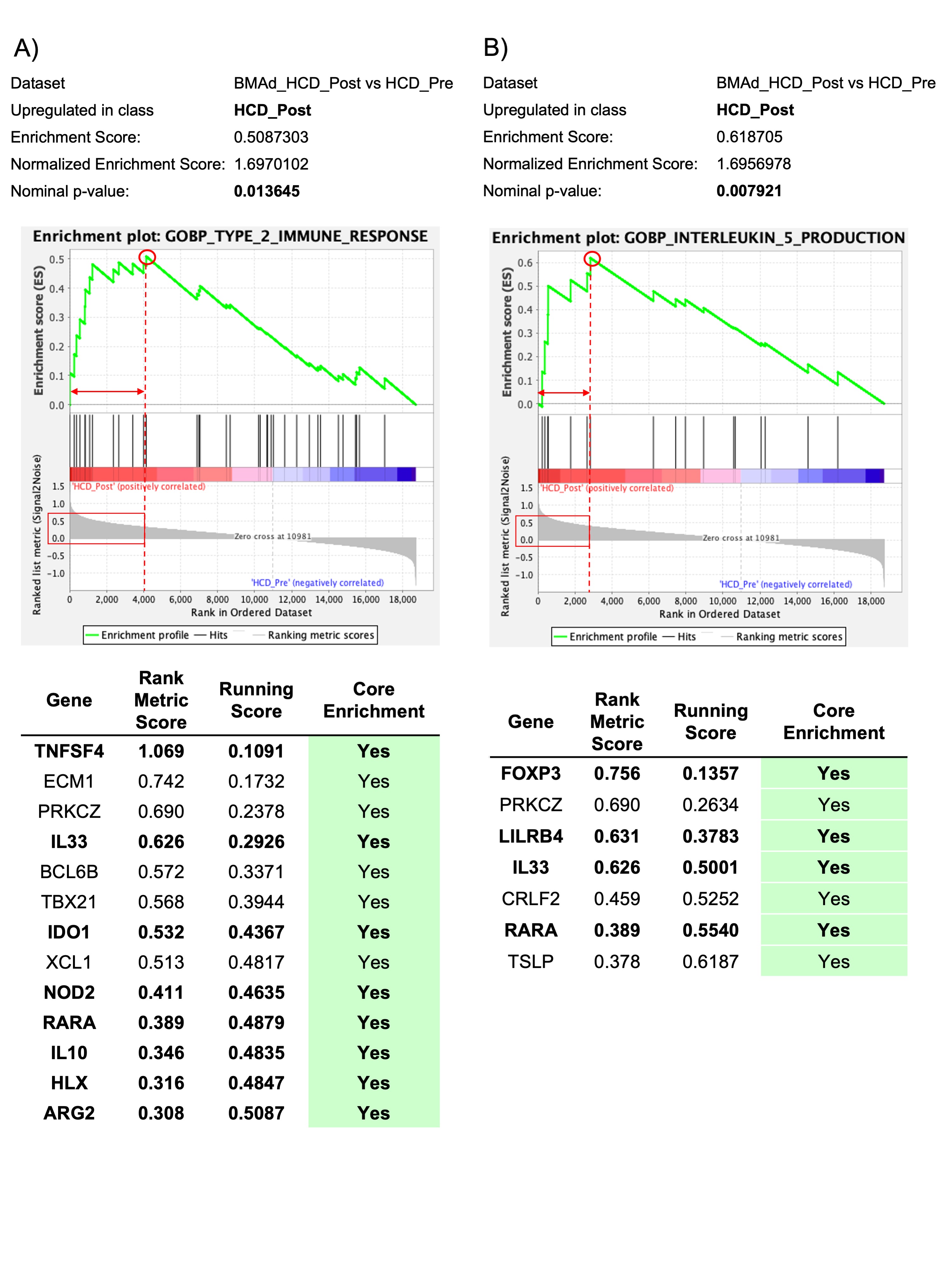

Supplement: Supplementary Figure 3 — Gene set enrichment analysis (GSEA) of upregulated genes post-HCD. (A) GSEA showed a significant (p-value < 0.05 and normalized enrichment score (NES) > 1.50) upregulation in the post:pre HCD for genes associated with the gene set Gene Ontology Biological Process (GOBP) Type 2 Immune Response. (B) GSEA showed a significant (p-value < 0.05 and normalized enrichment score > 1.50) upregulation in the post:pre HCD for genes associated with the gene set GOBP Interleukin 5 Production. The primary result is the enrichment score (ES), which reflects the degree to which a gene set is overrepresented at the top or bottom of a ranked list of genes (red circle). The leading-edge subset of a gene set is the subset of genes that contribute most to the ES (i.e., the core enrichment) (red arrow). The bottom portion of the plot shows the value of the ranking metric. The ranking metric measures a gene’s correlation with a phenotype. The value of the ranking metric goes from positive to negative as you move down the ranked list. A positive value indicates correlation with the phenotype profile (indicated with a red dotted line and red square). [file Image3.jpeg]

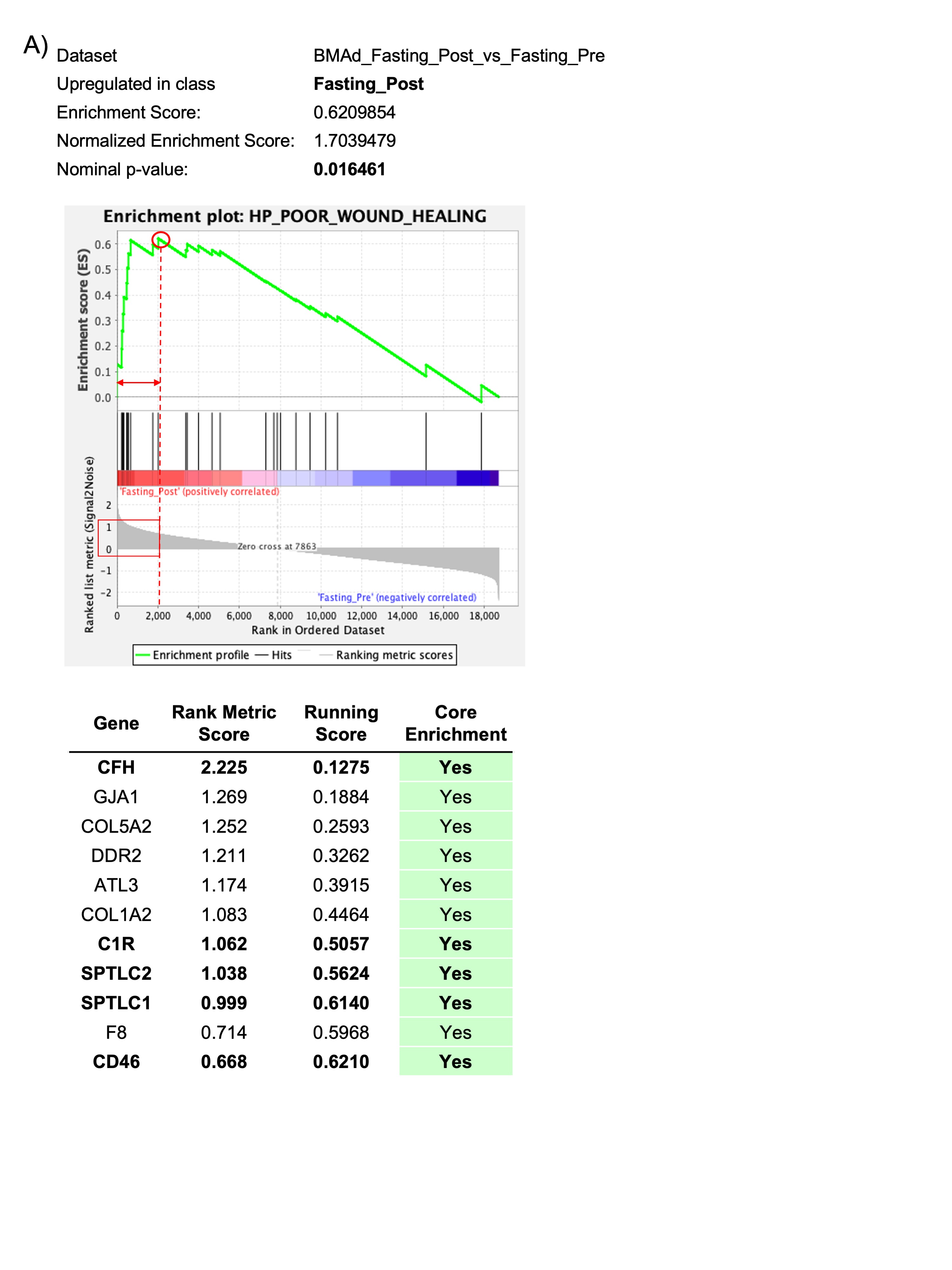

Supplement: Supplementary Figure 4 — Gene set enrichment analysis (GSEA) of upregulated genes post-fasting. (A) GSEA showed a significant (p-value < 0.05 and NES > 1.50) upregulation in the post: pre fasting for genes associated with the gene set Human Phenotype Ontology (HP) Poor Wound Healing. The primary result is the enrichment score (ES), which reflects the degree to which a gene set is overrepresented at the top or bottom of a ranked list of genes (red circle). The leading-edge subset of a gene set is the subset of genes that contribute most to the ES (i.e., the core enrichment) (red arrow). The bottom portion of the plot shows the value of the ranking metric. The ranking metric measures a gene’s correlation with a phenotype. The value of the ranking metric goes from positive to negative as you move down the ranked list. A positive value indicates correlation with the phenotype profile (indicated with a red dotted line and red square). [file Image4.jpeg]

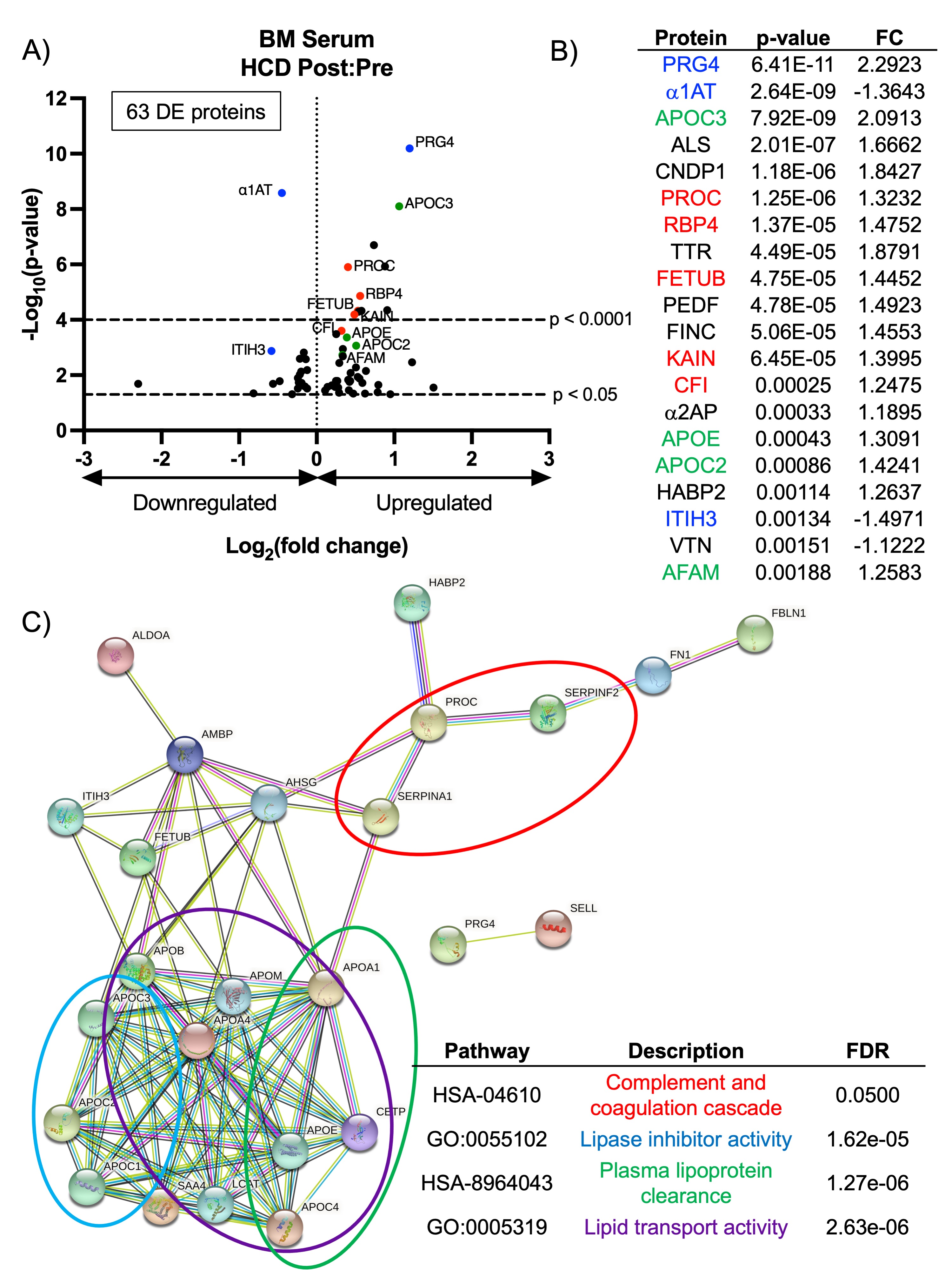

Supplement: Supplementary Figure 5 — Proteomic analysis of the BMS after HCD had a pro-inflammatory phenotype. (A) Volcano plot of the post:pre comparison after HCD (high nutritional effect) resulted in 63 DE proteins (p-value < 0.05 and FC > 1.0 or FC< -1.0) within the BMS. Proteins with a p-value < 0.0001 are above the second dotted line. (B) Top 20 proteins are listed in order of significance based on p-value. Proteins in blue represent are associated with anti-inflammatory properties, proteins in red are associated with inflammatory properties, and proteins in green are associated with metabolism; these proteins correlate to the genes on the volcano plot. (C) STRING analysis of 63 DE proteins. The network was created using an interaction score = 0.700 (high confidence) and by removing disconnected nodes. [file Image5.jpeg]

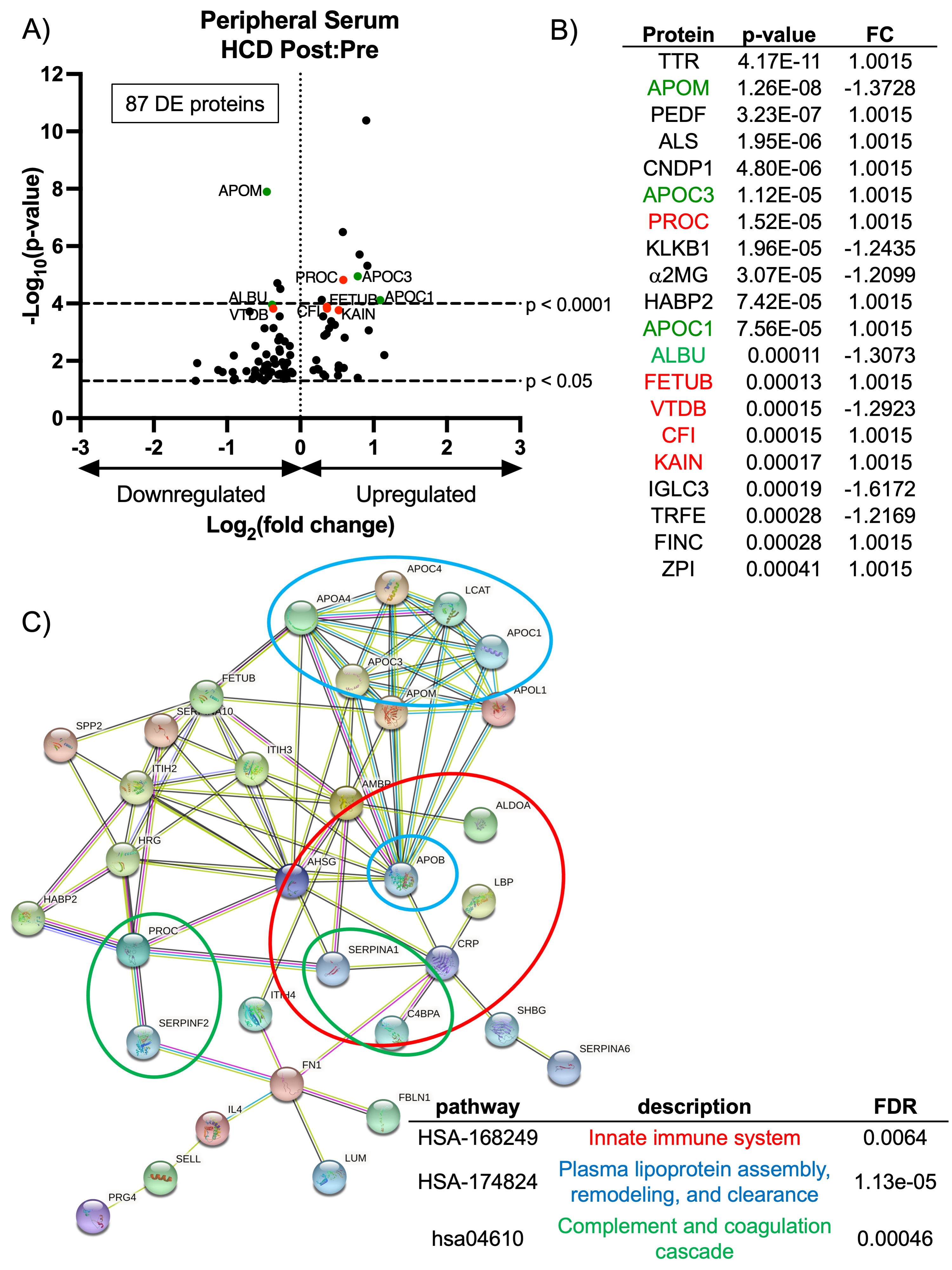

Supplement: Supplementary Figure 6 — Proteomic analysis of the PS after HCD showed a mild inflammatory phenotype. (A) Volcano plot of the post:pre comparison after HCD (high nutritional effect) resulted in 87 DE proteins (p-value < 0.05 and FC > 1.0 or FC< -1.0) within the PS. Proteins with a p-value < 0.0001 are above the second dotted line. (B) Top 20 proteins are listed in order of significance based on p-value. Proteins in red are associated with inflammatory properties, and proteins in green are associated with metabolism; these proteins correlate to the genes on the volcano plot. (C) STRING analysis of 87 DE proteins. The network was created using an interaction score = 0.700 (high confidence) and by removing disconnected nodes. [file Image6.jpeg]

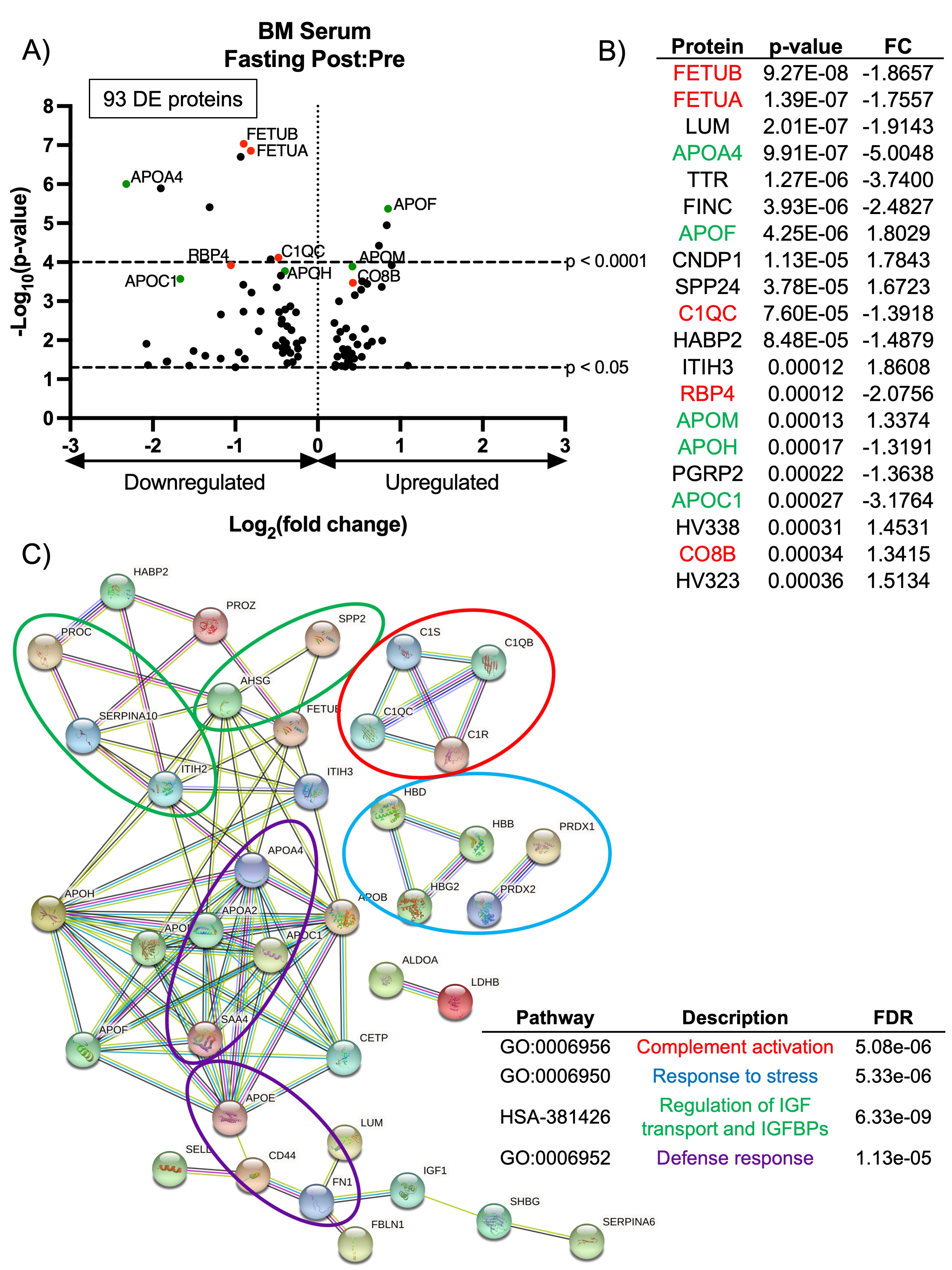

Supplement: Supplementary Figure 7 — Proteomic analysis of the BMS after fasting showed an anti-inflammatory phenotype. (A) Volcano plot of the post:pre comparison after fasting (low nutritional effect) resulted in 93 DE proteins (p-value < 0.05 and FC > 1.0 or FC< -1.0) within the BMS. Proteins with a p-value < 0.0001 are above the second dotted line. (B) Top 20 proteins are listed in order of significance based on p-value. Proteins in red are associated with inflammatory properties, and proteins in green are associated with metabolism; these proteins correlate to the genes on the volcano plot. (C) STRING analysis of 93 DE proteins. The network was created using an interaction score = 0.700 (high confidence) and by removing disconnected nodes. [file Image7.jpeg]

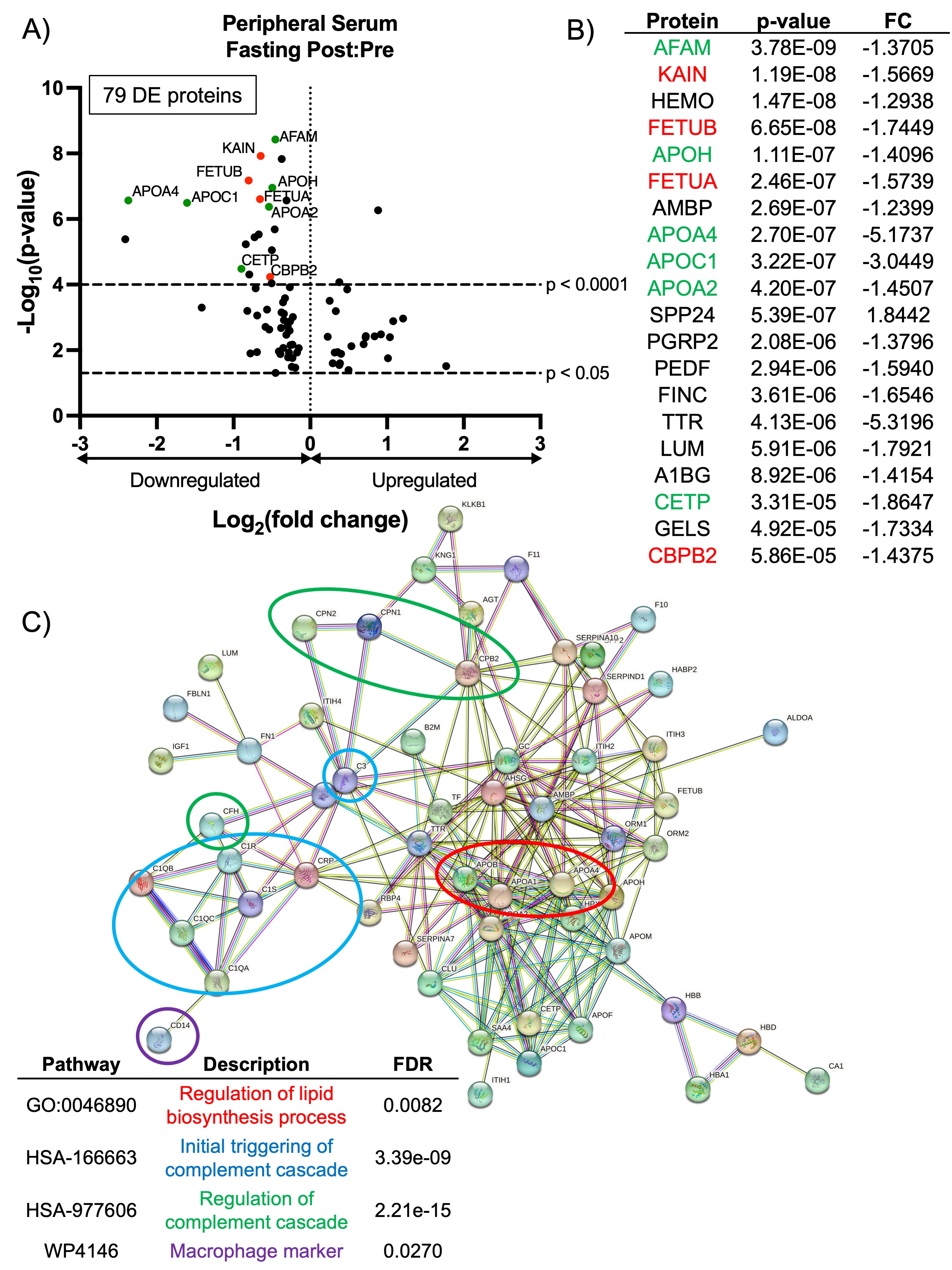

Supplement: Supplementary Figure 8 — Proteomic analysis of the PS after fasting showed a mild inflammatory phenotype. (A) Volcano plot of the post:pre comparison after fasting (low nutritional effect) resulted in 79 DE proteins (p-value < 0.05 and FC > 1.0 or FC< -1.0) within the PS. Proteins with a p-value < 0.0001 are above the second dotted line. (B) Top 20 proteins are listed in order of significance based on p-value. Proteins in red are associated with inflammatory properties, and proteins in green are associated with metabolism; these proteins correlate to the genes on the volcano plot. (C) STRING analysis of 79 DE proteins. The network was created using an interaction score = 0.700 (high confidence) and by removing disconnected nodes. [file Image8.jpeg]

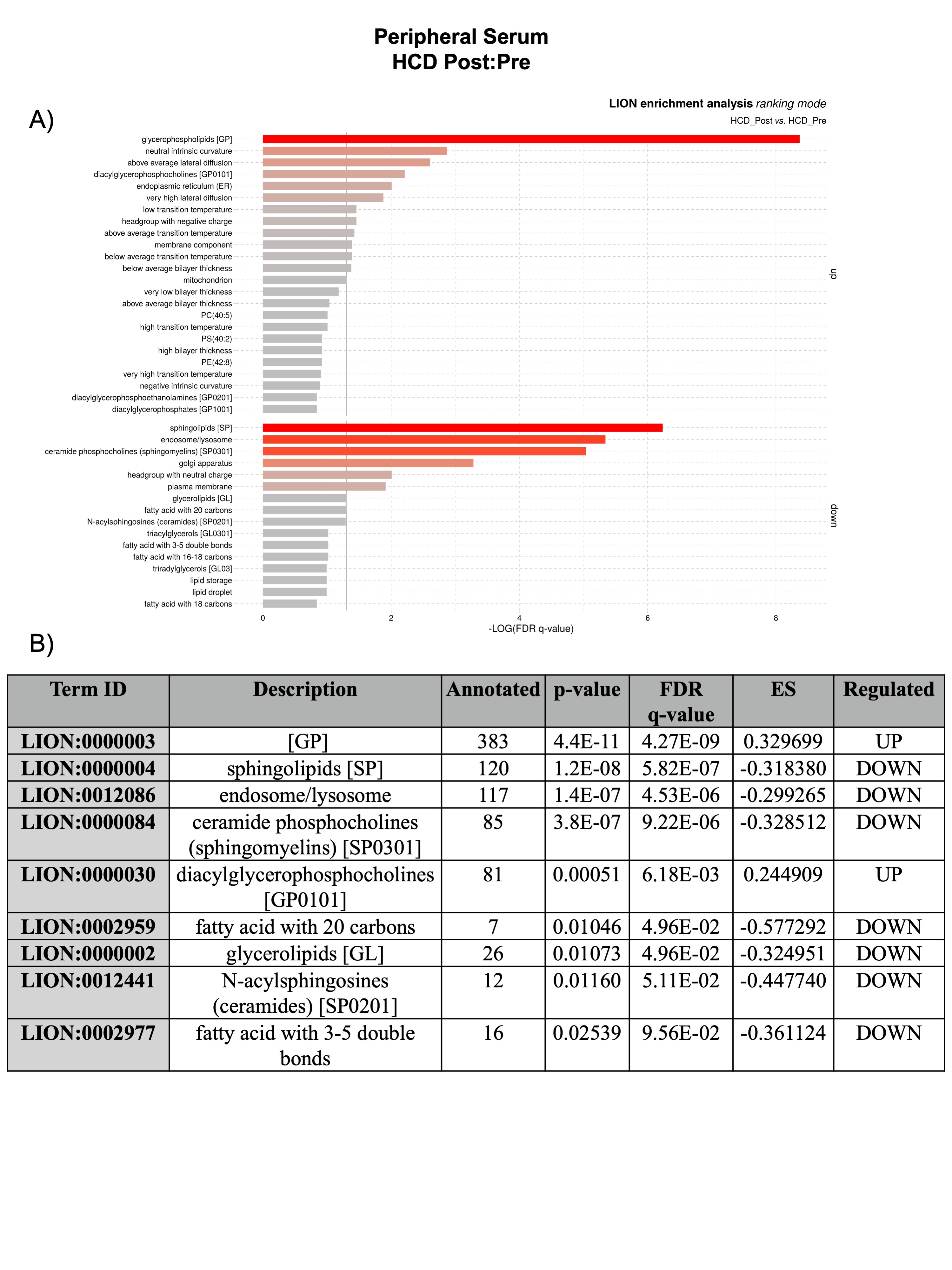

Supplement: Supplementary Figure 9 — LION enrichment analysis of the PS after HCD. (A) Lipid ontology (LION) enrichment analysis of positive and negative lipids (p-value < 0.05) from the PS lipidomic analysis after HCD (B). Top 9 LION terms based on p-value in the PS after HCD. [file Image9.jpeg]

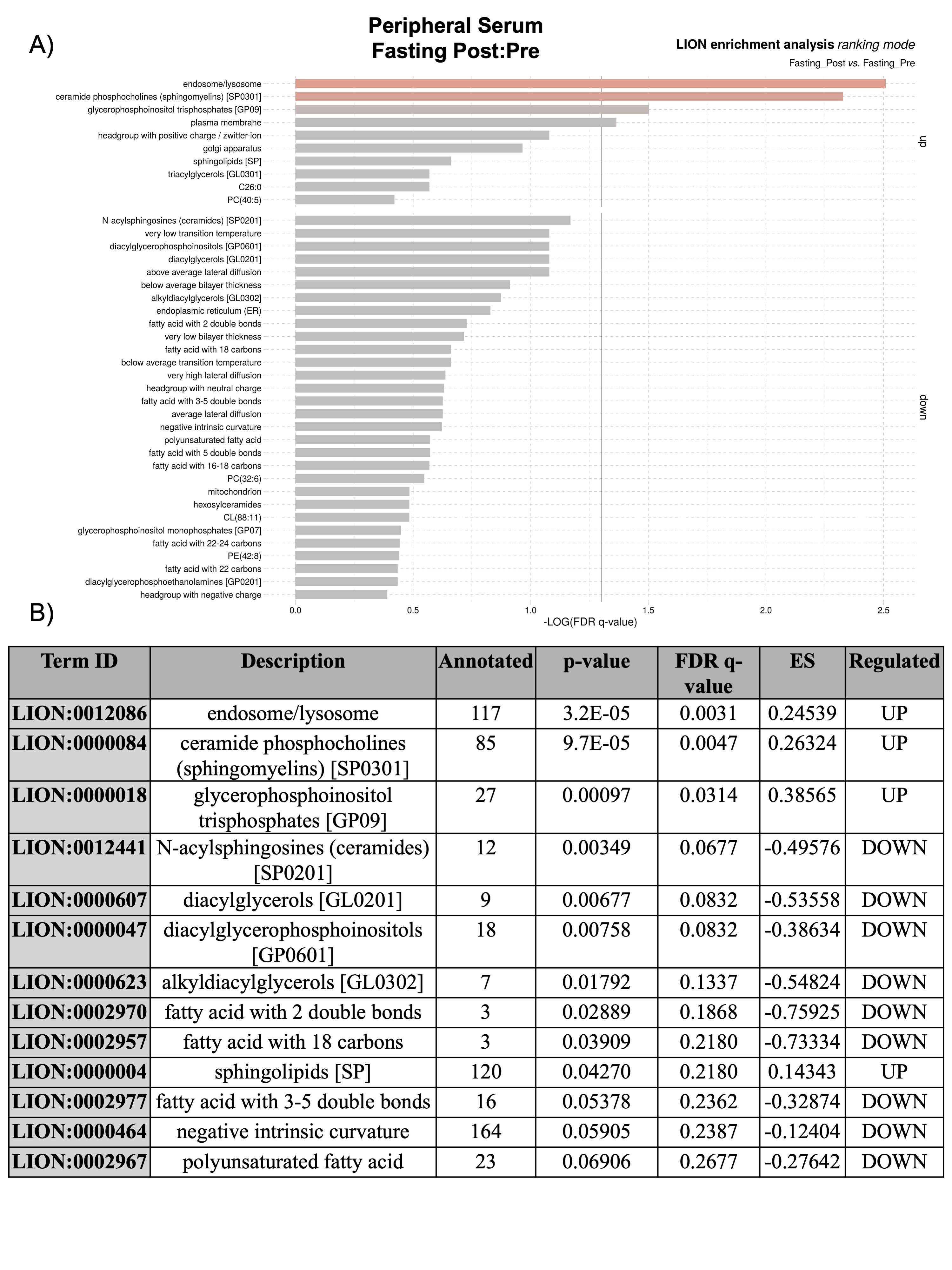

Supplement: Supplementary Figure 10 — LION enrichment analysis of the PS after fasting. (A) Lipid ontology (LION) enrichment analysis of positive and negative lipids (p-value < 0.05) from the PS lipidomic analysis after fasting. (B) Top 13 LION terms based on p-value in the PS after fasting. [file Image10.jpeg]
